# Cooperation and social organization depend on weighing private and public reputations

**DOI:** 10.1038/s41598-024-67080-z

**Published:** 2024-07-16

**Authors:** Matteo Cavaliere, Guoli Yang, Carsten K. W. De Dreu, Jörg Gross

**Affiliations:** 1 Department of Physics, Informatics and Mathematics, University of Modena and Reggio Emilia, Modena, Italy; 2 Department of Big Data Intelligence, Advanced Institute of Big Data, Beijing, 100195 China; 3 Faculty of Behavioral and Social Sciences, University of Groningen, Groningen, The Netherlands; 4 Faculty of Economics and Business, University of Groningen, Groningen, The Netherlands; 5 Behavioral Ecology and Sociobiology Unit, German Primate Center, Leibniz Institute for Primate Research, Göttingen, Germany; 6 Department of Psychology, University of Zurich, Zurich, Switzerland

**Keywords:** Evolutionary games, Cooperation, Information integration, Decision making, Evolutionary theory, Complex networks

## Abstract

To avoid exploitation by defectors, people can use past experiences with others when deciding to cooperate or not (‘private information’). Alternatively, people can derive others’ reputation from ‘public’ information provided by individuals within the social network. However, public information may be aligned or misaligned with one’s own private experiences and different individuals, such as ‘friends’ and ‘enemies’, may have different opinions about the reputation of others. Using evolutionary agent-based simulations, we examine how cooperation and social organization is shaped when agents (1) prioritize private or public information about others’ reputation, and (2) integrate others’ opinions using a friend-focused or a friend-and-enemy focused heuristic (relying on reputation information from only friends or also enemies, respectively). When agents prioritize public information and rely on friend-and-enemy heuristics, we observe polarization cycles marked by high cooperation, invasion by defectors, and subsequent population fragmentation. Prioritizing private information diminishes polarization and defector invasions, but also results in limited cooperation. Only when using friend-focused heuristics and following past experiences or the recommendation of friends create prosperous and stable populations based on cooperation. These results show how combining one’s own experiences and the opinions of friends can lead to stable and large-scale cooperation and highlight the important role of following the advice of friends in the evolution of group cooperation.

## Introduction

In a wide range of group-living animals, humans included, cooperators pay a cost to create benefits for others while defectors pay no cost and create no social benefits^1–4^. As defectors avoid the cost of cooperation and take advantage of others’ cooperation, they can more easily spread in the population and cooperation quickly deteriorates^4,5^. This can be prevented when cooperators rely on how partners behaved in the past and cooperate with those who cooperated previously (viz. direct reciprocity^6–9^). In addition, or alternatively, cooperators can rely on reputation^10^ that can be adjusted depending on the employed strategies of the interacting individuals^11,12^. Reputation can also be socially transmitted, for example through gossip^13,14^, to decide to cooperate or defect. Especially in larger groups, gossip or ‘public information’ allows for the evolution of cooperation through indirect reciprocity^15^. Depending on the veracity of gossip and public reputation-information, indirect reciprocity can maintain cooperation to different degrees^16–30^.

Previous works (e.g.,^18,22^) have extensively investigated scoring rules that assign reputation based on the action of a “donor” and the reputation of a “receiver”. These works led to eight rules that have been shown to stabilize cooperation through indirect reciprocity^22^ and rely on the ability to observe the actions of others in the population and apply shared norms on how to assign reputation. Deviating from this approach, in this paper we investigate a scenario where social norms are not defined and actions are based on personal affinity or enmity towards third-parties, sharing opinions.

Specifically, when deciding whether to cooperate with another agent, cooperators value the opinion of another agent to the extent that they had good experiences with this agent^31^. In this scenario, we, hence, assume that observing actions poses challenges (i.e., no unambiguous public image score is present) but exchanging opinions is straightforward and decisions to cooperate with an agent are made by weighting the experiences of other agents (public reputation information) against one’s own experiences (private reputation information) with the potential partner.

The fact that cooperators can use private and more or less veracious public reputation information raises two open and interrelated questions about the evolution of cooperation and social organization. First, private and public information can give contradictory signals. For example, whereas private information can indicate that a partner can be trusted, public information may suggest the partner most likely defects. This raises the question of how cooperation and social organization evolve when individuals differentially prioritize private and public information and signals are aligned versus contradictory and weighed differently. Second, whereas private information is reliable but perhaps based on few prior interactions, public reputation information is potentially based on more interactions, integrating experiences of ‘friends’—those that have cooperated with the individual in the past—and ‘enemies’—those that defected with the individual. Cooperators, however, may be purely ‘friend-focused’, relying only on information from those who cooperated with them in the past. Friend-focused individuals more likely cooperate with ‘a friend of a friend’ and not with ‘an enemy of a friend’. Alternatively, cooperators can also be ‘enemy-focused’ and, additionally, cooperate with an ‘enemy of an enemy’—partners who defected on those who defected on the individual^31^. Compared to friend-focused agents, these ‘Heider’ agents more likely cooperate with others and build broader networks, yet they may also mistake partners who are unconditional defectors for (conditional) cooperators and hence get exploited more often than friend-focused agents^32–39^. Especially when public information is prioritized, grounding cooperation in friend-focused or Heider heuristics can have important yet presently unknown consequences for the evolution of cooperation and social organization.

We address these open questions on the evolution of cooperation and social organization by studying a model in which agents use (i) different reputation heuristics (e.g., a ‘friend of a friend is a friend’) and (ii) differentially weigh recommendations by others with private opinions about one’s interaction partner^31,40^. Our results reveal how different weighing of public and private information creates different patterns of cooperation and free-riding even in the absence of noise or strategic misinformation (i.e., related to the literature on gossip veracity). Furthermore, results reveal a crucial role of friendships and the opinions of friends for the resilience of group cooperation, and how the interplay of private and public opinions alongside different reputation heuristics influence social organization and network polarization.

### Computational model

For our analysis, we assume a population of agents that interact in two-person social dilemmas in which cooperation is individually costly yet beneficial to the partner. When the partner cooperates, the agent’s relationship to the partner is strengthened (i.e., relationship score *s* increases by a quantity *r*; with − 1 ≤ s ≤ 1). If the partner defects, the relationship is weakened (i.e., relationship score *s* decreases by *r*) (*Materials and Method*). For future interactions, such private information can (i) help to distinguish cooperators from defectors and establishing reciprocal relationships but (ii) is limited by the amount of information that can be gathered. Especially in larger populations, the challenge for agents is to correctly distinguish cooperators from defectors and accordingly decide whether to cooperate or to defect, even if reliable private information is not available (i.e., due to the limited number of direct interactions). In such scenarios, it becomes worthwhile to obtain information on the trustworthiness of interaction partners from other agents in the population (e.g., through gossip and information sharing, i.e., the exchange of experiences and evaluations) and integrate such information based on simple reputation heuristics.

**Figure 1:**
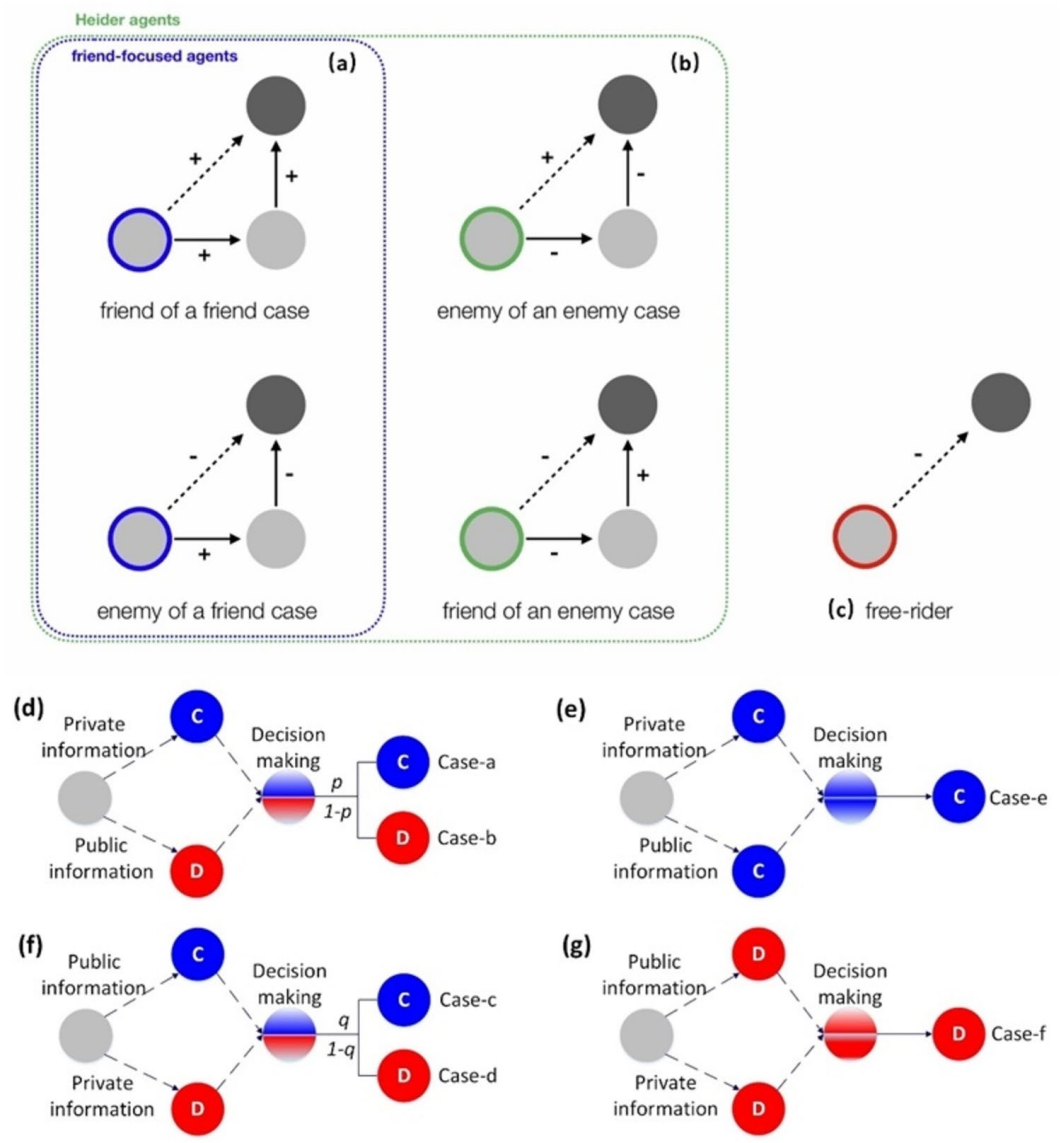
Three types of agents: (**a**) friend-focused agents (nodes with blue border), (**b**) Heider agents (nodes with either green or blue borders) and (**c**) defectors (node with red border), where ‘friendship’ is indicated by the symbol ‘ + ’ and ‘enmity’ is indicated by the symbol ‘-’. The potential interaction partner is the dark-colored node. Friend-focused agents (a) compute a score of public information (shown by the dashed lines) by considering the opinions from only friends (nodes connected with links ‘ + ’) which would indicate to cooperate with ‘a friend of a friend’ and withhold cooperation from ‘an enemy of a friend.’ Heider agents (**b**) compute a score of public information by considering the opinions from both friends and enemies (nodes connected with links ‘ + ’ and ‘ − ’) which would indicate to avoid cooperation when their partner is ‘a friend of an enemy’ and cooperate when the partner is ‘an enemy of an enemy’. Defectors always defect, and all links outgoing are hence marked ‘ − ’. Agents’ ultimate decision to cooperate or not is based on private and public information (grey node in **d**–**g**). When they provide consistent indications, the decision is made accordingly (**e**,**g**). When they provide conflicting signals (**d**,**f**), decisions are based on weighing parameters *p* and *q* (blue states denote cooperation; red states denote defection).

Following^40^, we assume that agents ground their assessment of their partner on a combination of public and private information^6^. An agent obtains private information by considering its own relationship towards a new partner based on direct past experiences. Private information is reliable but limited since the agent may have had only few or no interactions with the partner. Hence, in our model agents also rely on indirect, public information—the opinions of others towards the partner. Public information can compensate for the lack of (direct) private experiences but can also be misleading. For example, an enemy of an enemy may be a friend, and cooperates, or a defector who exploits. Accordingly, the rule-of-thumb agents use for public information matters, and we incorporated in our model (a) friend-focused agents denoted by *F*, and (b) Heider agents denoted by *H*^31,39^ (Fig. 1). Friend-focused agents decide whether to cooperate or defect based on the opinion of others with which the agent has a positive relationship with (‘friend heuristic’)—they more likely cooperate with ‘a friend of a friend’ and withhold cooperation from ‘an enemy of a friend.’ Heider agents consider the opinions from both friends (using the friendship heuristic outlined above) and ‘enemies’^31,39^—they more likely withhold cooperation when their partner is ‘a friend of an enemy’ and cooperate when the partner is ‘an enemy of an enemy’ (enemy heuristics). We compare these two agent-types that, in principle, are willing to cooperate, to agents that exploit other cooperators (‘defectors’ denoted by *D*).

Our model finally assumes that agents combine public and private information^6,40^ to establish whether to cooperate or defect (Fig. 1d–g). When private and public information provide a consistent signal (e.g., the partner can be trusted), the agent will use the signaled strategy (Fig. 1e,g). However, when private and public information provide a contradictory signal (Fig. 1d,f), the parameters *p* and *q* are used to decide whether to cooperate. Specifically, if private information indicates to cooperate and public information indicates to defect, then the agent will cooperate with probability *p* (case-a in Fig. 1d) and defect with probability 1 − *p* (case-b in Fig. 1d). If, in contrast, private information indicates to defect and public information indicates to cooperate, then the agent cooperates with probability *q* (case-c in Fig. 1f), and defects with probability 1 − *q* (case-d in Fig. 1f). Parameters *p* and *q* thus capture the degree to which agents’ cooperation relies on private information (own experiences with the partner) or public information (the aggregated opinion of other agents in the population, weighted by the agents’ relationship with them based on the agent’s reputation heuristics). For details on the decision-making of the agents see *Methods and Materials (*section on *Action Choice).*

Larger values for *p* and smaller values for *q* correspond to a stronger reliance on private information over public information; larger values for *q* and smaller values for *p* prioritize public over private information. In our model, high values for both *p* and *q* lead to decisions where defection is chosen only when both public and private information signal to defect, and cooperation is chosen when either public or private information signals to cooperate. We refer to this scenario as ‘high trustiness’, since agents ‘trust’ their private experiences to cooperate (even when public information recommends otherwise) or ‘trust’ public information to cooperate (even when private information recommends otherwise) and only choose to defect when both, private and public information, signal to defect. Low values for both *p* and *q*, on the other hand, lead to decisions where cooperation is chosen only when both public and private information are aligned and signal to cooperate (‘low trustiness’ in either signal). Note that defectors do not rely on either private or public information since they simply always defect (*Methods and Materials*).

## Results

To disentangle the interplay of (i) how public information is gathered (friend-focused agents vs. Heider agents) and (ii) how the integration of private and public information (parameters *p* and *q*) influences cooperation, we first analyze homogenous populations (only friend-focused or Heider agents), then consider pairwise competition (two types of agents), and, lastly, analyze the most complex environment: a population composed of all three types of agents.

### Heider heuristics and prioritizing public information lead to polarization

In homogenous populations with only friend-focused (*F*) or only Heider (*H*) agents there is no threat of defector invasions, and simulations can reveal how successful different reputation heuristics are in establishing cooperation depending on the integration of private and public information. Because *F* and *H*-agents are, in principle, willing to cooperate with others, in the best-case scenario, a population emerges in which every agent is connected and cooperates with every other agent. Yet depending on how information from others is integrated and the degree to which decisions are driven by private or public information, agents may mistakenly defect with a cooperator or mutually defect because their ‘friend-circle’ or private experiences recommend doing so.

**Figure 2:**
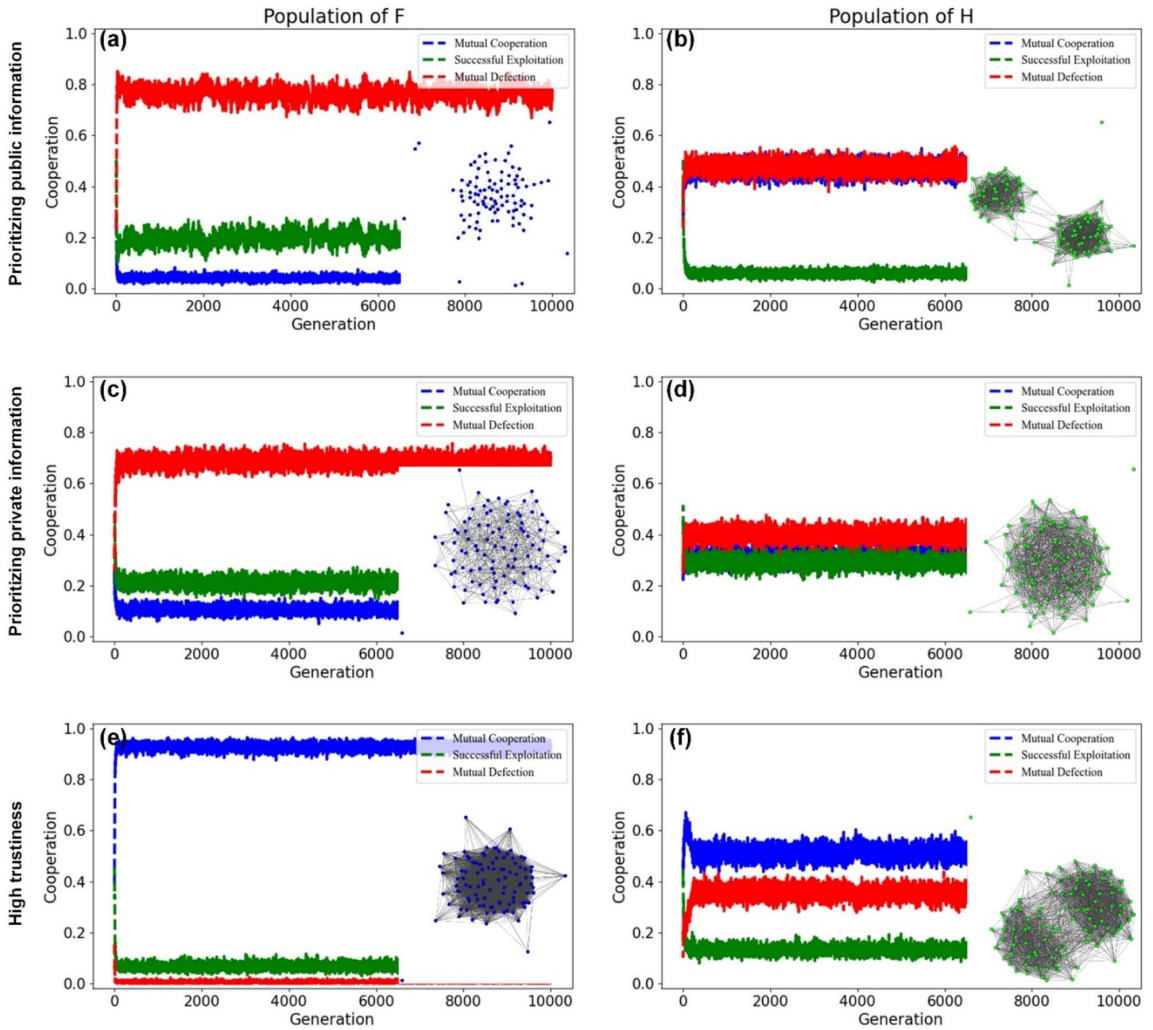
Population dynamics for homogeneous populations. The snapshots and trajectories in the left panels show a population of friend-focused agents (F; blue nodes), and those in the right panels show a population of Heider agents (H; green nodes). The figures illustrate the proportion of mutual cooperation (C–C, blue curves), exploitation (C–D, green curves), and mutual defection (D–D, red curves). Polarization emerges in the population of H agents when public information strength is high. Cooperation substantially increases in the case of high trustiness for a population of F agents. The plots are obtained by using a simulation composed by 1 × 10^4^ steps for *p* = 0.2 and *q* = 0.8 (first row), *p* = 0.8 and q = 0.2 (second row), *p* = 0.8 and *q* = 0.8 (third row). The size of the population is N = 100, iteration frequency is i = 10, mutation rate is µ = 0.01, and the amount of reputation change is r = 0.3.

Figure 2 illustrates the evolution and snapshots of the population of *F*-agents (left panels) and that of *H*-agents (right panels). We code actions as mutual cooperation (*C–C*), exploitation (*C-D*), and mutual defection (*D-D*; *Methods and Materials*). In these homogenous populations, simulations quickly reach a stationary state, yet the degree of mutual cooperation depends on the weighing of private and public information. When public information is prioritized (*p* = 0.2 and *q* = 0.8), *F*-agents create populations with few mutually cooperative connections (Fig. 2a). In contrast, when private information is prioritized (*p* = 0.8 and *q* = 0.2), *F*-agents develop more mutually cooperative relationships, but the population is marked by low interconnectedness and mutual defection being the dominant interaction outcome (Fig. 2c). Only in the case of high trustiness (i.e., agents cooperate either when private information or public information indicates to do so), *F*-agents develop large-scale cooperation (Fig. 2e).

In homogenous populations of *H*-agents, we observe a markedly different pattern. *H*-agents create higher levels of mutual cooperation, both when prioritizing public (*p* = 0.2 and *q* = 0.8; Fig. 2b) and when prioritizing private information (*p* = 0.8 and *q* = 0.2; Fig. 2d). At the same time, however, when prioritizing public information (*p* = 0.2 and *q* = 0.8, Fig. 2b), and when weighing private and public information equally (*p* = *q* = 0.8),* H*-agents polarize into two distinct groups with strong cooperation within and no cooperation between groups (Fig. 2f). This polarization is not observed for *F*-agents and can be attributed to the enemy heuristic that lead *H*-agents to classify partners as either part of one’s friendship circle (i.e., ‘an enemy of an enemy is my friend’) or not (i.e., ‘a friend of an enemy is an enemy’). Accordingly, Heider heuristics can lead to higher levels of cooperation than friend-focused heuristics, but this comes with stronger polarization of the population into distinct groups. Results are robust when the value of *r* (governing the amount of reputation change after each interaction; *Methods and Materials*) is modified as long as it exceeds a minimal value (SI Fig. S1 and S2).

### Heider heuristics and prioritizing public information facilitate defector invasions

Our next step is to evaluate which of the two types of strategies survive against defectors, and how they compete against each other. We conduct evolutionary-based simulations in which agents interact pairwise using the default parameters of population size *N* = 100, iteration frequency *i* = 10, mutation rate *µ* = 0.01, and the amount of reputation change *r* = 0.3. We first consider the possible scenarios of pairwise interactions with defectors (*F*-agents vs. defectors, FD; and *H*-agents vs. defectors, HD; *Methods and Materials*) to evaluate which agent-type better resist defectors under different weighing of private and public information (parameter *p* and *q*). Besides the frequencies of the three types of actions (as above), we further calculate population instability by using the standard deviation of agents’ population size (i.e., the change in the agent composition across time), prosperity as the average payoff of the population, and the number of communities in the population based on positive links that connect nodes with positive relationship values (*Methods and Materials*).

For the maintenance and proliferation of cooperation, an agent’s strategy needs to (i) correctly identify and isolate defectors and (ii) correctly identify (and cooperate with) other cooperators. To avoid (i) invasions of defectors, agents need to avoid cooperating with a defector (‘successful isolation’), and to increase (ii) overall cooperation and build strongly interconnected networks, cooperators need to cooperate with other agents that are cooperating with them and avoid defection with others that are, in principle, willing to cooperate (‘pairwise successful cooperation’). Based on these categorizations, we analyze how often *F*- and *H*-agents decide to cooperate or defect towards defectors (with which they ‘should’ defect) and other agents of their own type (with which they ‘should’ cooperate), accordingly.

**Figure 3:**
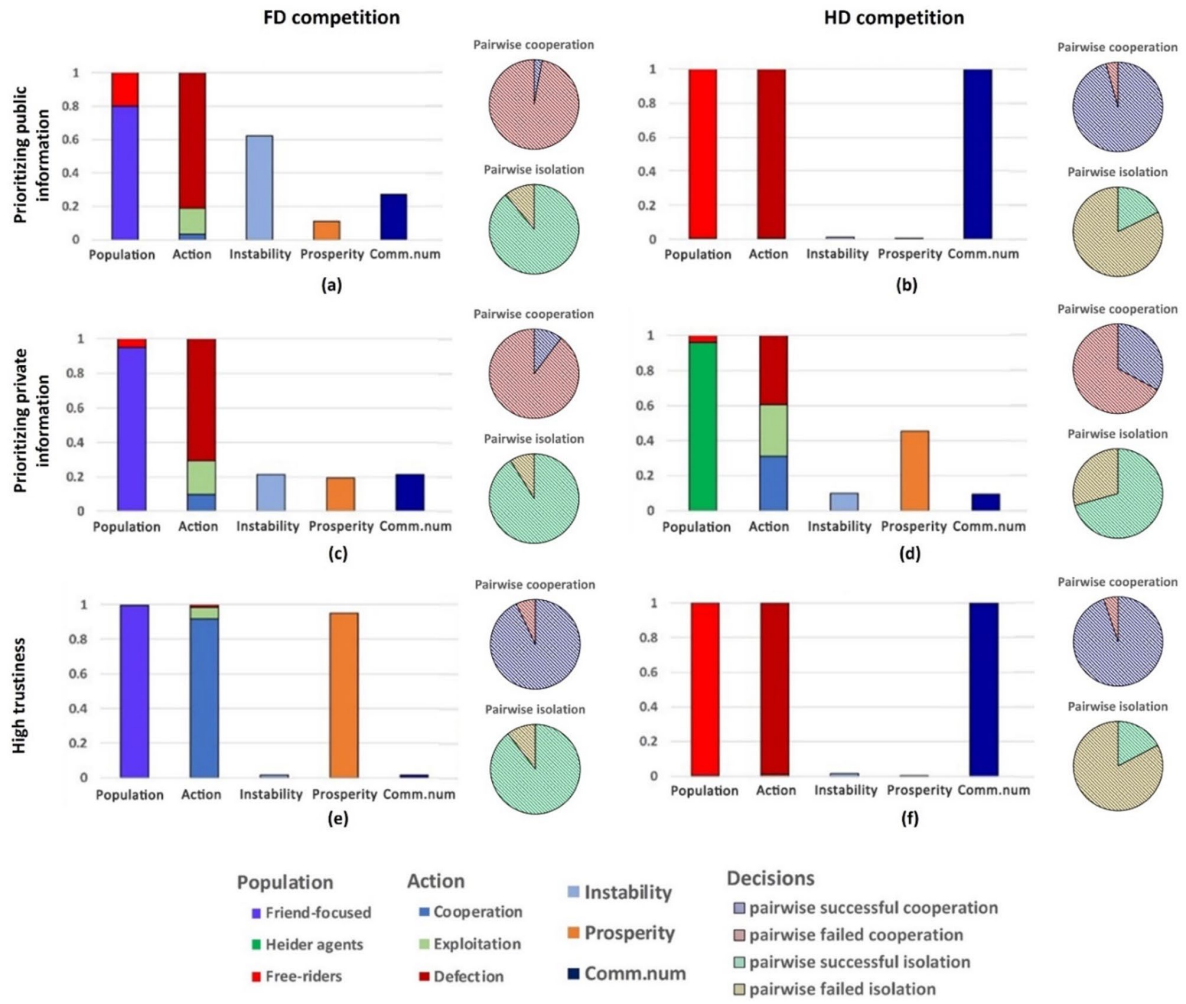
Heider agents (H) outcompete defectors (D) only in the case of prioritizing private information. Defectors, on the other hand, are outcompeted by friend-focused agents (F) in all three scenarios. Pairwise competitions of FD and HD are obtained by starting in a population with 50% of the two strategies considered. On the right of each considered scenario, we plot the rate of pairwise successful cooperation (C–C) and pairwise failed cooperation (C–D, D–C and D–D) considering pairs of same-type agents (namely F-and-F or H-and-H), and the rate of pairwise successful isolation (D–D) and pairwise failed isolation (C–D) when meeting defectors (see colors in the legend). Averages are obtained by running 10 independent simulations and each lasts 5 × 10^6^ steps.

We first consider the proportion of *C–C* actions when two same-type agents meet, and successful isolations as the proportion of *D-D* actions when a *F*- or *H*-agent meets a defector. This reveals that friend-focused agents can survive against defectors when they rely on public information (Fig. 3a), on private information (Fig. 3c) or exhibit high trustiness (Fig. 3e). Since these F-agents only trust their own opinion or that of agents they already established a positive relationship with, they do not risk making connections to, and cooperating with defectors. In essence, the way friend-focused agents integrate reputation information prevents them from mistakenly cooperate with defectors—successful isolation of defectors in FD interactions exceeds by some margin that in HD interactions (Fig. 3a,c; *SI* Fig. S3).

While friend-focused agents are successful in isolating defectors, correctly identifying other cooperators depends on how they integrate information. When considering only public or private information, *F*-agents often fail to recognize other cooperators of their own type, leading to disproportionally few pairwise cooperations, and small and scattered friendship circles with limited prosperity (Fig. 3a,c). Only when both public and private information are considered, pairwise cooperation for friend-focused agents is high, and prosperous and stable populations with high interconnectedness emerge (Fig. 3e).

A different pattern emerges for Heider agents. When public information takes precedence over private information, *H*-agents fail to reliably identify defectors and die out (Fig. 3b). In contrast, Heider agents that prioritize private information successfully isolate defectors (Fig. 3d; *SI* Fig. S3d). Regardless, however, there remains limited successful pairwise cooperation, with low prosperity (Fig. 3d; *SI* Fig. S3d). Combined, this points to a cooperation-prosperity tradeoff for Heider agents. To shield against free-riding, they lose their ability to create densely interconnected networks and often fail to cooperate with other Heider agents (Fig. 3d).

### Friend-focused agents dominate in high trustiness environments

**Figure 4:**
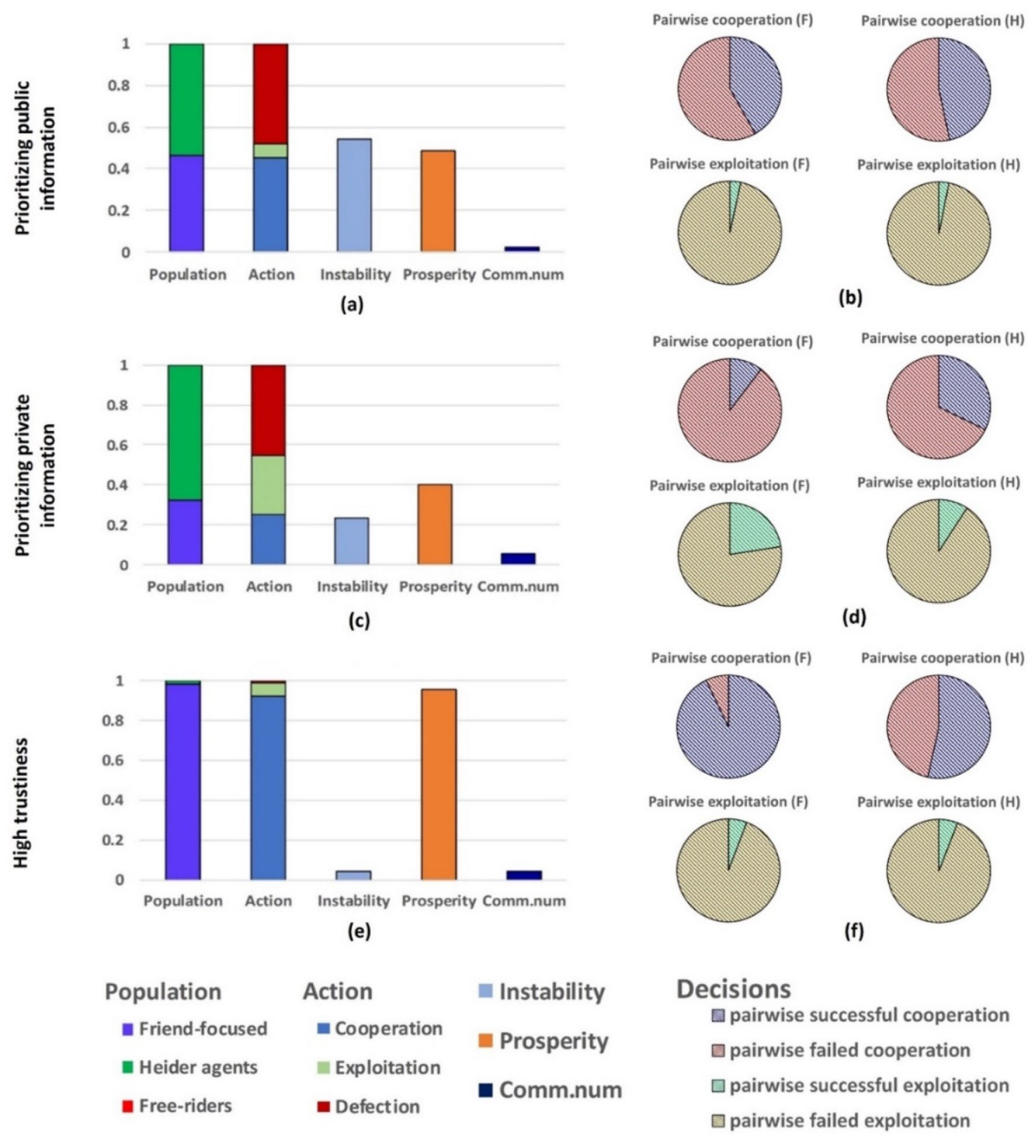
Friend-focused (F) agents outcompete Heider (H) agents in the case of high trustiness leading to a regime of highly cooperative, stable, connected, and prosperous populations. Pairwise competitions of FH are illustrated in the case of prioritizing public information (upper panels), prioritizing private information (middle panels) and high trustiness (lower panels), which are obtained by initializing a population with 50% of the two strategies considered. Statistical properties and the probability of decisions taken by friend-focused agents or Heider agents towards same and different type of agents are presented. Cooperation vs exploitation panels illustrate the proportions of pairwise successful cooperation (C–C) and pairwise failed cooperation (C–D, D–C and D–D) considering pairs of same-type agents, and the proportions of pairwise successful exploitation (D–C) and pairwise failed exploitation (C–C, C–D and D–D) between pairs of friend-focused and Heider agents (see colors in the legend). Averages are obtained by running 10 independent simulations and each lasts 5 × 10^6^ steps.

Considering interactions between Heider and friend-focused agents reveals that the two types of agents co-exist when either public or private information is prioritized (Fig. 4a,c). Heider agents are more often able to identify each other and to build cooperative relationships compared to friend-focused agents (Fig. 4b,d), giving them a slight competitive advantage over friend-focused agents (Fig. 4a,c; *SI* Fig. S4). However, due to the large proportion of pairwise failed cooperation between agents of the same type, neither *F*- nor *H*-agents form prosperous cooperative communities (Fig. 4b,d).

Results change when friend-focused agents cooperate either when their friends recommend cooperating, or their own past experiences tell them to do so (*p* = *q* = 0.8). In such a high trustiness environment, friend-focused agents dominate both defectors (Fig. 3e) and Heider agents (Fig. 4e).

As friend-focused agents successfully connect to cooperative friend-focused agents and, at the same time, limit cooperation with defecting friend-focused agents (Fig. 4f), they have a considerable advantage over Heider agents who more often defect against cooperative Heider agents (Fig. 4f). These two mechanisms behind the success of friend-focused agents reinforce each other under high trustiness, allowing them to connect to other cooperators (e.g., following recommendations by their friends) without risking being exploited by defectors. Heider agents invoking the enemy heuristic, in contrast, often fail to cooperate with other cooperative Heider agents (case-f in Fig. 1; *SI* Fig. S4f.) (for analyses covering the entire parameter space, see *SI* Fig. S5 and S6).

### Prioritizing public information leads to cycles of Heider and friend-focused dominance

**Figure 5:**
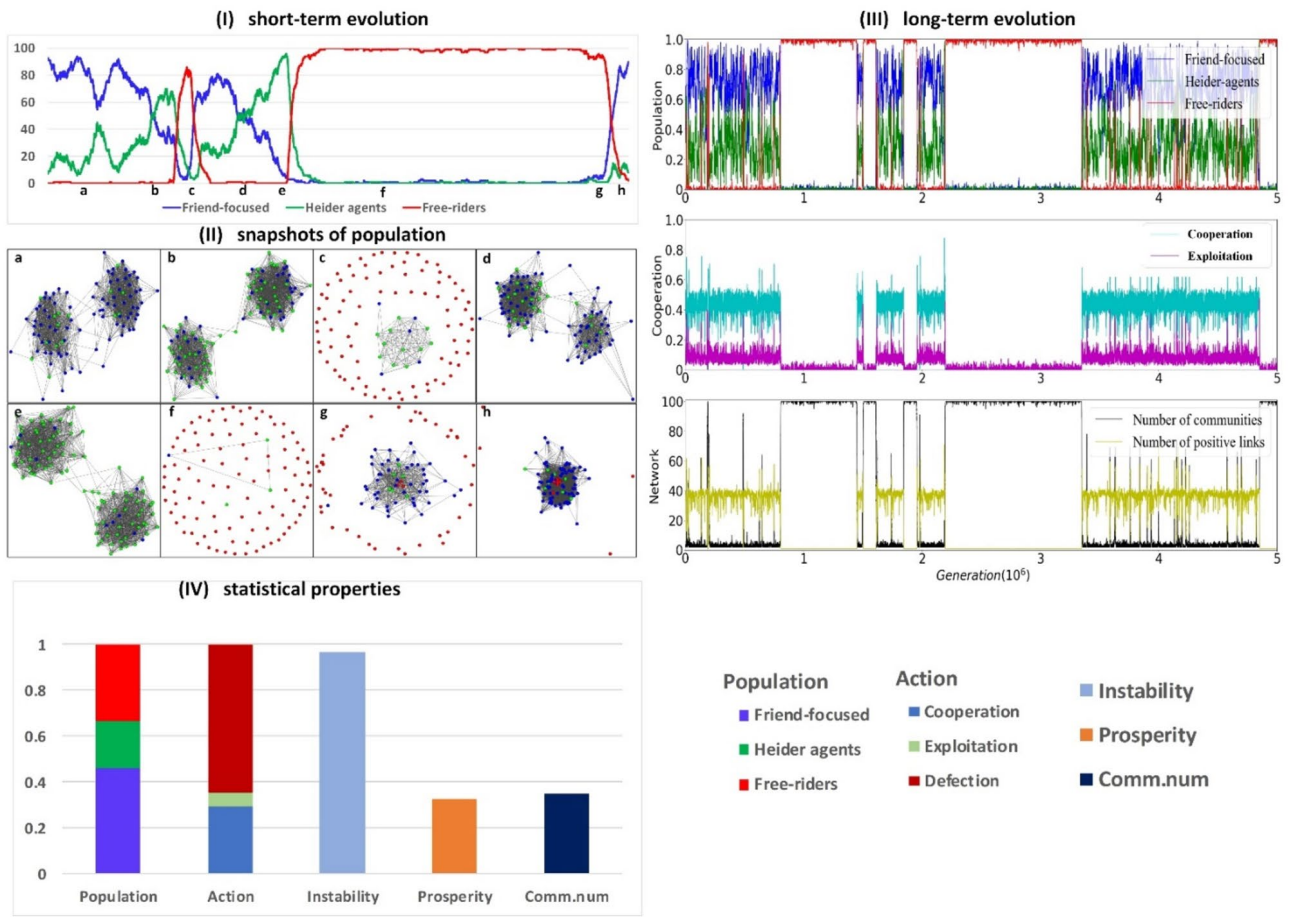
Evolutionary dynamics for prioritizing public information. The rise of Heider agents (green nodes) facilitates the invasion of defectors (red nodes), and the population becomes polarized before being invaded by defectors. Growth of cooperation is generally associated with an increasing number of positive links and decreasing number of communities. The plots are obtained by using a simulation composed by 5 × 10^6^ steps for *p* = 0.2 and *q* = 0.8. The size of the population is N = 100, iteration frequency is i = 10, mutation rate is µ = 0.01 and the change of reputation is r = 0.3. The figure illustrates the proportion of friend-focused agents (blue curves), Heider agents (green curves), defectors (red curves), fraction of cooperation (cyan curves) and exploitation (magenta curves), the number of communities (black curves) and positive links (yellow curves). Panel IV shows the long-term average values of different types of agents, actions, instability, prosperity, and the number of communities (see colors in the legend).

**Figure 6:**
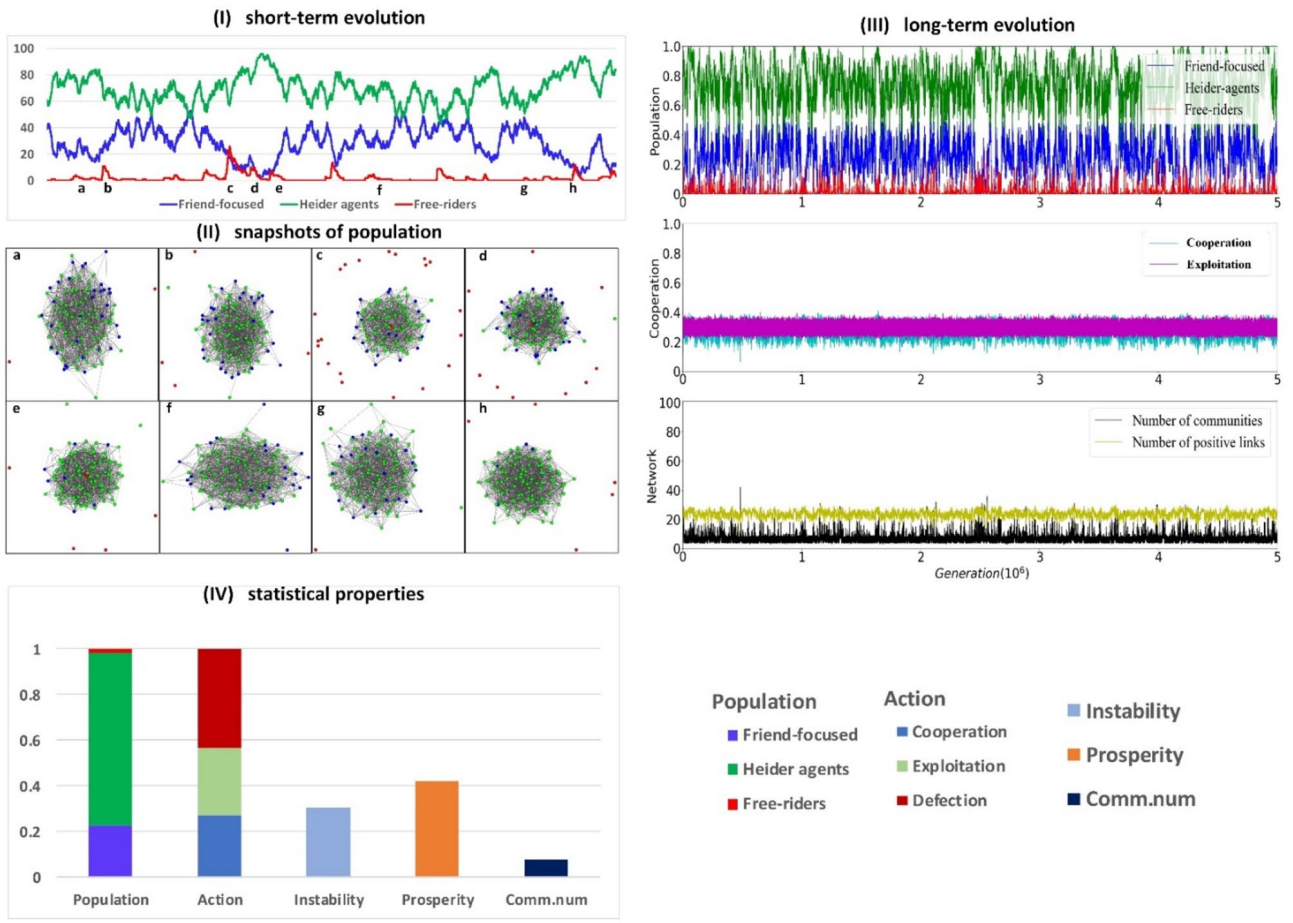
Evolutionary dynamics for prioritizing private information. Invasions by defectors are rare. At the same time, there are lower levels of cooperation and higher levels of exploitation than in the case of prioritizing public information. The population is mainly composed of Heider agents with a minority of friend-focused agents with a low number of positive links. The plots are obtained using a simulation composed of 5 × 10^6^ steps for *p* = 0.8 and *q* = 0.2. The size of the population is N = 100, iteration frequency is i = 10 and mutation rate is µ = 0.01 and the change of reputation is r = 0.3. In the figure, we illustrate the proportion of friend-focused agents (blue curves), Heider agents (green curves), defectors (red curves), fraction of cooperation (cyan curves) and exploitation (magenta curves), the number of communities (black curves) and positive links (yellow curves). Panel IV shows the long-term average values of different types of agents, actions, instability, prosperity, and the number of communities (see colors in the legend).

**Figure 7:**
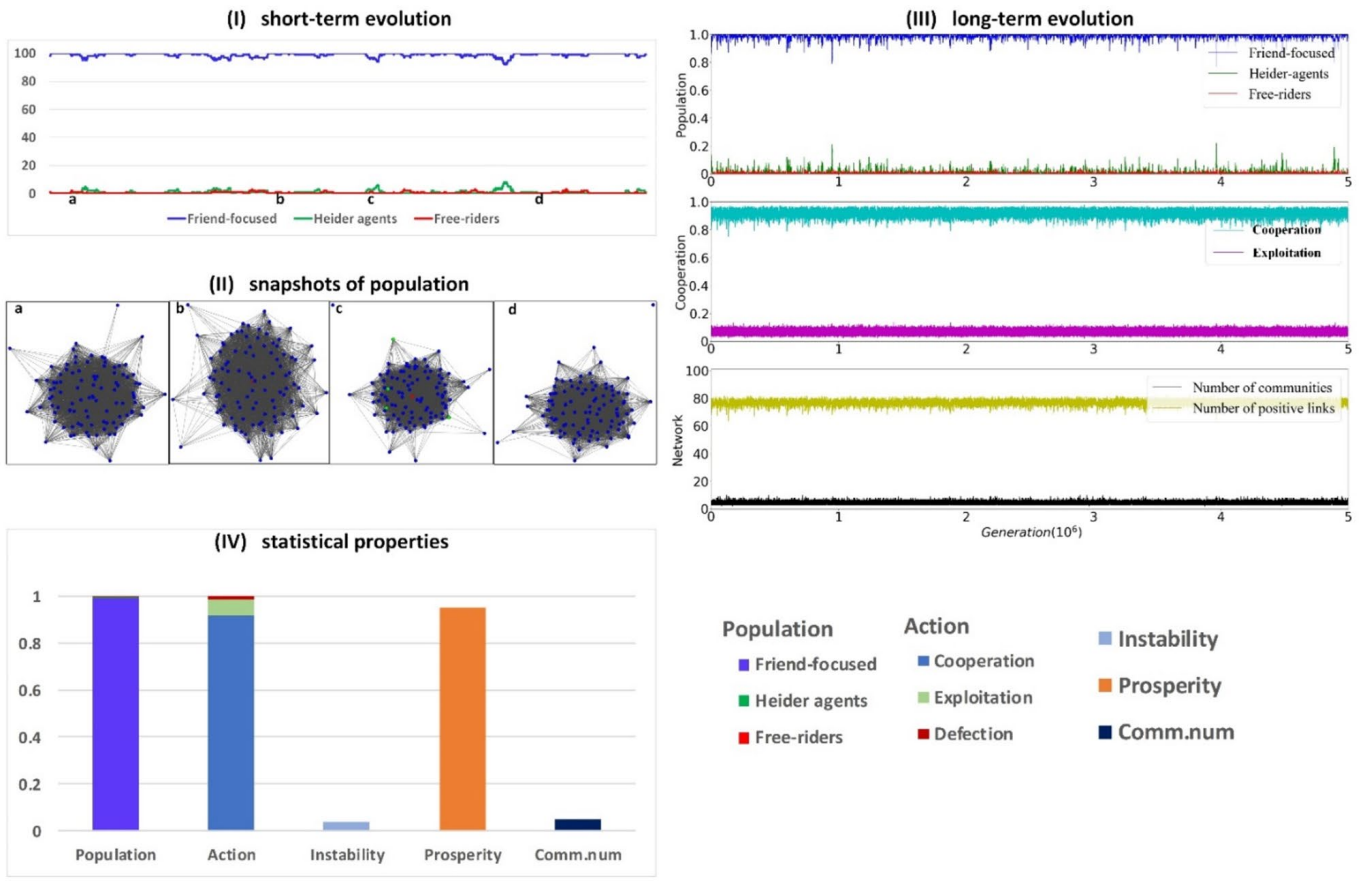
Evolutionary dynamics for high trustiness. Well-connected friend-focused agents are dominant in the population that is characterized by stable and high levels of cooperation and prosperity and no invasions of Heider agents or defectors. The plots are obtained using a simulation composed of 5 × 10^6^ steps for *p* = 0.8 and *q* = 0.8. The size of the population is N = 100, iteration frequency is i = 10 and mutation rate is µ = 0.01 and the change of reputation is r = 0.3. In the figure, we illustrate the proportion of friend-focused agents (blue curves), Heider agents (green curves), defectors (red curves), fraction of cooperation (cyan curves) and exploitation (magenta curves), the number of communities (black curves) and positive links (yellow curves). Panel IV shows the long-term average values of different types of agents, actions, instability, prosperity, and the number of communities (see colors in the legend).

Having analyzed the dynamics of homogenous populations and pairwise competitions of the different types of agents, we conclude by examining how the integration of private and public information modulates cooperation and social organization in populations comprised of all three types of agents. Our simulations start with a population of N agents engaging in a two-person prisoner’s dilemma where all three types of agents (*H*, *F* and *D*) can be present (*Methods and Materials*). Figures 5, 6, 7 illustrate the short-term (with 1 × 10^4^ steps) and long-term (with 5 × 10^6^ steps) evolution of the population, and the long-term average values of population properties—the proportion of the different types of agents, the types of actions, the number of communities, and population prosperity and instability.

When public information is prioritized (*p* = 0.2 and *q* = 0.8), we observe cycles of invasions for the three types of agents. Populations dominated by friend-focused agents are invaded by Heider agents (Fig. 5.II and Fig. 4a), and the rise of Heider agents (green nodes) in turn facilitates the invasion of defectors (red nodes), followed by the collapse of cooperation and social structure (cyan curves in Fig. 5.III and Fig. 3b). In fact, the emergence of Heider agents (Fig. 5.I) and concomitant polarization within populations (Fig. 5.II) can be taken as a ‘predictor’ of the impending break-down of cooperation and spreading of defectors (Fig. 5.I), with populations becoming fragmented (Fig. 5.II). When the population is full of defectors (Fig. 5.IIf), the network is maximally fragmented into isolated nodes and the number of communities increases as positive links between communities disappear (Fig. 5.III). After such a temporary invasion of defectors, communities of friend-focused agents can form again (Fig. 2a and Fig. 5.IIh) due to their dominance over defectors (Fig. 3a).

When private information is prioritized (*p* = 0.8 and *q* = 0.2), Heider agents (green curves) are most prevalent (Fig. 6.I; and Fig. 4d) and defectors rarely invade (red lines in Fig. 6.I and III). While population instability and cycles of cooperation, polarization, and invasions by defectors disappear, the rate of mutual cooperation remains low (Fig. 6.III and 6.IV) and the population is only loosely connected (Fig. 6.II). Hence, prioritizing private information helps to avoid defector invasions but cooperation remains limited (Fig. 3c,d), as Heider agents do not create as many social (i.e., positive) ties as we observed when public information is prioritized (Fig. 6.III vs Fig. 5.III; and also Fig. 2b,d). In this scenario, the stability of population is achieved at the cost of small communities with fewer positive links, limited mutual cooperation and more exploitation (Fig. 6.IV).

When both private and public information is equally and strongly incorporated (*p* = *q* = 0.8), we observe stable and well-connected populations (Fig. 7.II, and Fig. 2e) dominated by friend-focused agents (Fig. 7.I and 7.III). Strong reliance on both public and private information thus creates stable and prosperous communities of friend-focused agents that are robust against invasions by both Heider agents and defectors (Fig. 7.I and 7.IV; and Fig. 3e, Fig. 4g).

More generally, increasing the values of *p* and *q* reveals a shift from the dominance of Heider agents to that of friend-focused agents (SI Fig. S7 and S8). As a robustness check on the observed dynamics, we investigate the evolutionary dynamics by systematically varying the public information strength *q* in the case of weak private information p (SI Fig. S9 and S10) and strong private information *p* (SI Fig. S11 and S12). When prioritizing public information, the population becomes polarized just before the invasion of defectors, which fragments the population into isolated nodes upon which friend-focused agents start emerging and the population recovers to a mixed configuration (SI Fig. S11 and S12). Also, we confirm that the dominance of Heider agents is generally associated with high levels of defection, leading to limited prosperity. Conversely, the dominance of friend-focused agents is generally associated with high cooperation, low defection, and high prosperity (SI Fig. S13-S14). Results in SI Fig. S15 suggest the presence of *thresholds*: Friend-focused agents dominate the population when the strength of private information *p* is above ≈ 0.4 and *q* above ≈ 0.5 while Heider agents dominate the population when the strength of public information *q* is below ≈ 0.3. Also, Heider agents win in the case of prioritizing private information, and friend-focused agents win in the scenario of high trustiness, even when the population contains two extra strategies which represent limited variants of the Heider agents (SI Fig S16-S17; SI Table 1).

## Discussion

In systems where individual decisions to cooperate are based on personal past experiences and weighted ‘opinions’ of friends and enemies, global cooperation, emerging population structures and compositions are highly dependent on how private and public information is integrated. Our agent-based simulations show, first, that the presence of Heider agents is beneficial for the entire community when agents prioritize either private or public information. In this case, Heider agents can help the population to increase cooperation rates, connectivity and prosperity. Having Heider agents in the population leads to higher average prosperity compared to populations that only harbor friend-focused agents. However, relying strongly on public information comes at the risk of polarization followed by defector invasions as Heider agents are weak against defectors when trust in public information is strong. The population is then ‘rescued’ by friend-focused agents which, in contrast, are very successful against defectors. As Heider agents dominate friend-focused agents, in the case of prioritizing private information and, to a less extend, in the case of prioritizing public information, this leads to a perennial co-existence of these two types of strategies, alternated with temporary defector invasions, more striking in the case of high trust in public information. Population instability and cycles of cooperation, polarization, and invasions by defectors drastically diminishes when agents prioritize own opinions (private information) over those of others, but cooperation rates remain low in this case.

To create large, interconnected communities and achieve high levels of cooperation, low instability and high prosperity, large trust in the own opinion or that of friends is needed. When agents cooperate either when their own experiences or the public opinion tell them to, agents emerge that only take the opinion of their friends into account. In this scenario, friend-focused agents not only dominate the population by avoiding exploitation from defectors, they are also able to connect to other cooperators by following the advice of their friends even if their private opinion tells them otherwise. Such full-trust agents that rely on the own opinion or follow the ‘recommendation’ of friends when in doubt are not invaded by Heider agents, can correctly identify and isolate defectors, and create interconnected cooperative communities. These results highlight the importance of friendships and following the advice of friends for the resilience of cooperation clusters.

In the absence of full trust, populations need Heider agents to create interconnected communities, yet this comes at the cost of frequent polarization and cooperation breakdowns. Enemy heuristics thus are a double-edged sword that allow to make connections to new agents (e.g., trust an ‘enemy of an enemy’) and thereby increase population-wide cooperation levels but also invite defector invasions (e.g., when the ‘enemy of an enemy’ is a defector). Taken together, trust in the personal opinion and following friends’ opinions only (even when personally in doubt) is the most successful strategy to create well-connected communities marked by high cooperation that also crowd out more polarizing heuristics (i.e., ‘enemy of my enemy is my friend’). This strategy successfully decreases the risk of ‘misleading’ gossip by only considering recommendations of friends.

Findings shed light on the how social structures can dynamically evolve^41^ through a combination of private and public information^40^. Our model can also be seen as a possible framework to integrate two core mechanisms (that to date have been largely investigated in isolation) underlying the evolution of cooperation: direct and indirect reciprocity^42–47^. Importantly, in our model, the mechanism to update the reputation of not-cooperating individuals is on the recipient side, setting it apart from other reputation score models in which public reputation scores exist in the population. Also, how the reputation score is taken into account crucially depends on the heuristics employed: for friend-focused agents, if the recipient is not in the friend-circle, the potential loss of reputation is outside of one’s own group. So, if an agent x defects against y, this can be irrelevant from the perspective of x, if y is not part of the friendship circle (as x has no public reputation score). For Heider-agents having a bad reputation (in some agent’s eyes) could even be advantageous since an ‘enemy of an enemy’ may be more likely to cooperate, subsequently. Given these assumptions, our findings can be particularly helpful to understand the social organization and polarization of populations where such heuristics are present, there is some system of sharing opinions (i.e., gossip), and individuals balance private experiences with the opinions of others in the absence of clear social norms and public reputation scores^31,41^. Results can also shed light on group-structured populations in which agents are particularly motivated to uphold a good reputation within their group but are not concerned with upholding a positive reputation when interacting with agents outside of the friendship circle.

Future work could extend the current model by considering information availability and authenticity (i.e., related to gossip veracity) or strategic miscommunication of private opinions. Importantly, our results show that mistakes (e.g., cooperating with a defector) as well as polarization dynamics can emerge even without introducing noise or strategic miscommunication (e.g., intentionally spreading false gossip). Also, allowing the evolution of more general strategies (parameters p and q which are used to weight public and private information) could lead to additional insights on the evolutionary stability of the proposed mechanisms. While the current focus of the paper is to look at the edge-cases of (a combination of) friend and enemy heuristics for deriving reputation in combination with the strength of public/private information, another possibility would be to study a model with a continuous strategy-space obtained by using (two) continuous parameters defining the degree of friendship between two interacting individuals.

More generally, we believe that combining private and public information as a way to establish the reputation of individuals can be applied to many other types of scenarios, and at different scales of organization^5,48–52^. In essence, following one’s own opinion or that of friends, and cooperating even when these opinions differ, can yield stable and high levels of cooperation. On the flipside, following public opinion, even when private experiences tell otherwise, results in polarization and frequent breakdown of cooperation. In sum, results underscore the importance of friendships and the advice of friends for the emergence of large-scale cooperation and reliable social information transmission that complements personal experiences.

## Methods and materials

### Computational model

We consider the evolutionary dynamics in a population consisting of different agents. There are three types of agents: friend-focused agents, Heider agents and defectors^31^. Friend-focused and Heider agents can play ${\varvec{C}}$ (cooperate) or ${\varvec{D}}$ (defect) while defectors (i.e., cheaters or defectors) only play ${\varvec{D}}$.

In a population with size $$N$$, for an agent $$x$$, its payoff $$f(x)$$ is obtained by interacting with an opponent $$y$$ and is defined in the following way; If $$x$$ plays $$C$$ and $$y$$ plays $$C$$ as well, then $$f(x)=b-c$$. If $$x$$ plays $$C$$ and $$y$$ plays $$D$$, then payoff $$f(x)=-c$$. If $$x$$ plays $$D$$ and $$y$$ plays $$C$$, then payoff $$f(x)=b$$. If $$x$$ plays $$D$$ and $$y$$ plays $$D$$ as well, then payoff $$f(x)=0$$. This can be summarized in the payoff matrix which represents the Prisoner's Dilemma (with $$b >$$ $$c>0)$$.

1 $${ \begin{array}{c} \\ \varvec{\Pi=} \end{array}} \begin{array}{cc} & \begin{array}{cc} C& D\end{array}\\ \begin{array}{c}C\\ D\end{array}& \left(\begin{array}{cc}b-c& -c\\ b& 0\end{array}\right)\end{array}$$

The intuitive interpretation of the payoff matrix is that an agent that plays $C$ (cooperator) provides a benefit $b$ to their opponent, paying a cost $c$; an agent that plays $D$ (defector) pays no cost and gives no benefit.

As in^31^ the relationships between the agents are represented using an $N\times N$ matrix $\mathbf{S}$. The diagonal represents the relationship of every agent towards itself, which is set to 1. Each row ${\mathbf{m}}_{x}$ represents the relationship vector of $x$, i.e., its relationship towards every other agent in the population (including itself). Each column ${\mathbf{n}}_{x}$ represents the reputation vector of $x$, i.e., the relationship of every other agent towards $x$.

2 $$\mathbf{S}=\left[\begin{array}{cccc}1& {s}_{\text{1,2}}& \cdots & {s}_{1,N}\\ {s}_{\text{2,1}}& 1& \cdots & {s}_{2,N}\\ \vdots & \vdots & \ddots & \vdots \\ {s}_{N,1}& {s}_{N,2}& \cdots & 1\end{array}\right]$$

In a population of agents organized by the relationship matrix $\mathbf{S}$, the link from agent $x$ to agent $y$ is positive if ${s}_{x,y}>0$; otherwise if ${s}_{x,y}<0$, then the link from $x$ to $y$ is considered negative.

### Decision making and updates of reputations

Following^31^, each update step is composed by the following actions: (1) random matching, (2) action choice, (3) relationship update.

**(1) Random matching:** Repeatedly and randomly select and pair agents $x$ and $y$ in the population to play the prisoner's dilemma game, until all agents in the population have been paired.

**(2) Action choice:** When paired, agents choose the action to play (cooperate or defect) in the following way. If the agent $x$ is a defector, then always choose to defect. Otherwise, if the agent $x$ is friend-focused or Heider, then it chooses to cooperate depending on the reputation of the opponent. There will be two probabilities for the agent $x$ to make the decision regarding whether to cooperate or not with agent $y$.

Following^40^, agents take decisions by integrating public and private information^6^. The first probability ${p}_{p}(C)$ denotes the chance to cooperate with agent $y$ given the available private information.

3 $${p}_{p}(C)=1/\left(1+\text{exp}\left(-\beta \times {s}_{x,y}\right)\right)$$

where ${s}_{x,y}$ is the relationship (opinion) of agent $x$ towards agent $y$ (representing, in our model, the *private information*). The second probability ${p}_{q}(C)$ denotes the probability to cooperate with agent $y$ given the available public information.

4 $${p}_{q}(C)=1/\left(1+\text{exp}\left(-\beta \times r{s}_{x}(y)\right)\right.$$

where $r{s}_{x}(y)$ is $x$ 's reputation score towards $y$ (representing, in our model, the *public information*).

The reputation score (public information) $r{s}_{x}(y)$ is computed differently depending on the type of the agent (Heider vs. friend-focused agents^31^):

(i) If $x$ is friend-focused, then $r{s}_{x}(y)={\mathbf{m}}_{x}^{{{\prime}}}\times {\mathbf{n}}_{y}-{s}_{x,y}$, where ${\mathbf{m}}_{\mathbf{x}}$ is $x$ th row vector in the relationship matrix $\mathbf{S}$, and ${\mathbf{n}}_{y}$ is $y$ th column vector. ${\mathbf{m}}_{x}^{{{\prime}}}=\text{max}\left\{0,{m}_{x}\right\}$ indicates that the negative element in ${\mathbf{m}}_{\mathbf{x}}$ is replaced with 0 (i.e., opinions of agents with whom the deciding agent has a negative relationship with are ignored). So, $r{s}_{x}\left(y\right)$ is the weighted and aggregated product (minus the private information) based on the opinion of friends. Friend-focused agents, hence, only act upon the two friend-heuristics: ‘a friend of a friend is a friend’ and ‘an enemy of a friend is an enemy’ (proportional to s).

(ii) If $x$ is a Heider agent, then $r{s}_{x}(y)={\mathbf{m}}_{x}\times {\mathbf{n}}_{y}-{s}_{x,y}$, where ${\mathbf{m}}_{x}$ is the $x$ th row vector, and ${\mathbf{n}}_{y}$ is $y$ th column vector. The relationship score $r{s}_{x}(y)$ is thus the weighted and aggregated product (minus the private information ${s}_{x,y}$) based on the four relationship Heider heuristics^31^: A friend of a friend is a friend, an enemy of a friend is an enemy, a friend of an enemy is an enemy, an enemy of an enemy is a friend (proportional to s).

Probabilities ${p}_{p}(C)$ and ${p}_{q}(C)$ are then used by agent $x$ to determine whether to cooperate or not with agent $y$. Let $P$ and $Q$ be Boolean variables of whether or not the private and public information signal to agent $x$ to choose cooperation, respectively. If $P=$ True, then the private information signals to agent $x$ to cooperate, otherwise it signals to defect (probability of having $P=$ True is given by ${p}_{p}(C)$). Similarly, if $Q=$ True, then the public information signals to agent $x$ to cooperate, otherwise it signals to defect (probability of having $Q=$ True is given by ${p}_{q}(C)$).

The ultimate decision to cooperate or not is taken by agent $x$ by combining the indications offered by the public and private information signal^40^. Specifically, the agent decides to be a cooperator (C) or a defector (D) in the following way:

(i) If $P\wedge Q$, then $x$ decides to be $C$;

(ii) If $P\wedge \neg Q$, then $x$ decides to be $C$ with probability $p$ and to be $D$ with probability $1-p$.

(iii) If $\neg P\wedge Q$, then $x$ decides to be $C$ with probability $q$ and to be $D$ with probability $1-q$.

(iv) If $\neg P\wedge \neg Q$, then $x$ decides to be $D$.

where case (ii) and (iii) indicate the existence of a conflict between the signal provided by one’s private information and the one provided by the obtained public information. The conflict is solved using the parameters $p$ and $q$ which, as discussed in the main text, are used to control the strength of the private and public information, respectively.

**(3) Relationship updating:** Following^31^, if the agent is a defector, then its relationship to the others is set to be − 1. Else if agents $x$ and $y$ are either friend-focused or Heider agents, then they will update their relationship in the following way.

(i) If $x$ chooses $C$, and $y$ chooses $C$, then ${s}_{x,y}+=r,{s}_{y,x}+=r$;

(ii) If $x$ chooses $C$, and $y$ chooses $D$, then ${s}_{x,y}-=r,{s}_{y,x}+=0$;

(iii) If $x$ chooses $D$, and $y$ chooses $C$, then ${s}_{x,y}+=0,{s}_{y,x}-=r$;

(iv) If $x$ chooses $D$, and $y$ chooses $D$, then ${s}_{x,y}-=r,{s}_{y,x}-=r$.

where $r$ is the amount of relationship change. If ${s}_{x,y}>1$ then ${s}_{x,y}=1$; if ${s}_{x,y}<-1$, then ${s}_{x,y}=-1$. The diagonal of the relationship matrix is 1.

### Decision indications

Public and private information can provide both consistent and contradictory signals (e.g., one could indicate to cooperate, the other one to defect). There are 2 cases of consistent signals and 4 different cases of contradictory signals to be considered for an agent $x$ (that is not a defector) which we label in different ways.

(i) case-$a$ : private information indicates to cooperate, but public information indicates to defect, and finally agent $x$ chooses to cooperate;

(ii) case-$b$: private information indicates to cooperate, but public information indicates to defect, and finally agent $x$ chooses to defect;

(iii) case-$c$: private information indicates to defect, but public information indicates to cooperate, and finally agent $x$ chooses to cooperate;

(iv) case-$d$: private information indicates to defect, but public information indicates to cooperate, and finally agent $x$ chooses to defect.

(v) case-*e*: private information indicates to cooperate, and public information indicates to cooperate as well, and finally agent $x$ chooses to cooperate.

(vi) case-$f$: private information indicates to defect and public information indicates to defect as well, and finally agent $x$ chooses to defect.

### Evolutionary dynamics

Each $i$ update steps (i.e., iteration frequency), the population will evolve as in the Moran process. After the $i$ th iteration, the population will select one random agent that will adapt its strategy based on selection and mutation^31^:

(i) fitness-based selection with probability $1-\mu$: the new strategy will be the one already in the current population selected in a proportional manner to its fitness;

(ii) random-based mutation with probability $\mu$: the new strategy will be a random one according to the population composition.

In the population, the fitness of an agent is computed as ${e}^{\pi (x)}$, where $\pi (x)={\sum }_{t=\tau +1}^{t=\tau +i} {f}_{t}(x)$ is the sum of payoffs obtained by $x$ during the past $i$ iterations (from $\tau +1$ to $\tau +i$). Once an agent has adapted its strategy, it will reset its relationship vector ${\mathbf{m}}_{x}$ and the reputation vector ${\mathbf{n}}_{x}$ to be 0 except that ${s}_{x,x}=1$.

In the main text we consider three different scenarios: (i) Homogeneous population: where only one type of agent, either only Heider or only friend-focused agents, are present in the population; (ii) Pairwise competitions: Heider and friend-focused agents are present in the population, or Heider agents and defectors are present in the population, or Friend-focused agents and defectors are present in the population; and (iii) All types of agents: Heider agents, friend-focused agents and defectors are present in the population to compete and evolve. Case (iii) is the most general but complex scenario, and (i) and (ii) are special cases.

**(i) Homogenous Population.** This is a special case in which populations only consist of one agent type. In this case, there are no evolutionary dynamics since populations only harbor one strategy type. These simulations therefore can inform us about the emerging network structure and overall level of cooperation when agents are not competing with defectors (or the other cooperation type).

**(ii) Pairwise Competition.** Pairwise competition is a special case of the standard evolutionary dynamics where only two (out of the three) strategies are allowed in the population. Depending on how many types of agents are allowed to evolve in the population, ${\varvec{F}}$ denotes friend-focused agents, ${\varvec{H}}$ denotes Heider agents, and ${\varvec{D}}$ denotes defectors (i.e., defectors) agents. For example, $\mathbf{F}\mathbf{H}$ denotes a population where friend-focused and Heider agents compete against each other.

Pairwise successful cooperation $(PSC)$ is calculated by the proportion of $C-C$ actions when two same type agents meet, otherwise, the rest is defined by pairwise failed cooperation.

5 $$PSC=\frac{\#(C-C)}{\#(C-C)+\#(C-D)+\#(D-C)+\#(D-D)}$$

Pairwise successful isolation $(PSI)$ is calculated by the proportion of $D-D$ actions when a friend-focused agent or Heider agent meets a defector, otherwise, the rest is defined by pairwise failed isolation.

6 $$PSI=\frac{\#(D-D)}{\#(C-D)+\#(D-D)}$$

Pairwise successful exploitation $(PSE)$ is calculated by the proportion of $D-C$ actions when a friend-focused agent meets a Heider agent, otherwise, the rest is defined by pairwise failed exploitation.

7 $$PSE=\frac{\#(D-C)}{\#(C-C)+\#(C-D)+\#(D-C)+\#(D-D)}$$

As in the standard evolutionary dynamics, at each update step, the agents in the population make decisions and update their relationship towards others. Each $i$ update steps (i.e., iteration frequency), the population will evolve following a death-birth rule, where one random agent is selected to adapt its strategy to a new one present in the population based on selection and mutation (as outlined above).

(iii) Heider, friend-focused and free riding agents. This is the general case with the competition among Heider agents, friend-focused agents and free riders. When all three types of agents are in the population, any one strategy can be present in the population based on selection and mutation.

### Population composition

Let ${n}_{t}(F)$ be the number of friend-focused agents present in the population at step $t$; in a similar way, the number of defectors at step $t$ is denoted by ${n}_{t}(D)$ and the number of Heider agents at step $t$ is denoted by ${n}_{t}(H)$. Then we can compute the average number of different agents present during the evolutionary dynamics.

$${\mu }_{F}=\frac{\sum_{t=1}^{T} {n}_{t}(F)}{T}, {\mu }_{H}=\frac{\sum_{t=1}^{T} {n}_{t}(H)}{T}, {\mu }_{D}=\frac{\sum_{t=1}^{T} {n}_{t}(D)}{T}$$

where $T$ is the total number of steps constituting a single simulation.

### Cooperation, exploitation, defection

Let $${n}_{t}(Coop)$$ be the number of mutual cooperative actions $$(C-C)$$ at step $$t$$; in a similar way, the number of exploitative actions $$(C-D$$ or $$D-C)$$ at step $$t$$ is denoted by $${n}_{t}(Exp)$$ and the number of mutual defective actions $$(D-D)$$ at step $$t$$ is denoted by $${n}_{t}(Def)$$. We can compute the average number of different actions during the evolutionary dynamics.

$${\mu }_{\text{Coop }}=\frac{\sum_{t=1}^{T} {n}_{t}(Coop)}{T}, {\mu }_{\text{Exp}}=\frac{\sum_{t=1}^{T} {n}_{t}(Exp)}{T}, {\mu }_{\text{Def}}=\frac{\sum_{t=1}^{T} {n}_{t}(\text{ Def })}{T}$$

where $T$ is the total number of steps of a single simulation.

### Instability, prosperity, communities

The instability of the population can be computed using the standard deviation of agents' size during the evolutionary dynamics, which is calculated as follows.

$${\sigma }_{F}=\sqrt{\sum_{t=1}^{T} {\left({n}_{t}(F)-{\mu }_{F}\right)}^{2}/T}, {\sigma }_{H}=\sqrt{\sum_{t=1}^{T} {\left({n}_{t}(H)-{\mu }_{H}\right)}^{2}/T}, {\sigma }_{D}=\sqrt{\sum_{t=1}^{T} {\left({n}_{t}(D)-{\mu }_{D}\right)}^{2}/T}$$

where $T$ is the total number of steps of a single simulation, ${\mu }_{F},{\mu }_{H}$ and ${\mu }_{D}$ are the average number of different types of agents.

Overall population instability is the sum of all three types of agents, which can be calculated as:

8 $$\sigma ={\sigma }_{F}+{\sigma }_{H}+{\sigma }_{D}$$

The prosperity is computed by the average payoff of the population. Let ${f}_{t}(x)$ be the payoff obtained by $x$ at step $t$, then the prosperity is calculated as:

9 $$Pr=\sum_{t=1}^{T} \sum_{x} {f}_{t}(x)/NT$$

The structure of the population is determined by the positive links, which are used to form connected communities. The number of positive links in a population is the sum of the positive relationship values, i.e.,

10 $${N}_{\text{pos-link}}=\sum_{x,y} {s}_{x,y}\times \epsilon \left({s}_{x,y}\right)$$

where $\epsilon (i)=1$ if $i>0$, and $\epsilon (i)=0$ if $i\le 0$. The average number of positive links in a population is calculated as ${N}_{\text{pos-link }}/N$.

Let ${cn}_{t}$ be the number of connected communities at step *t*, then the average number of communities (i.e. *Comm.num)* is calculated as:

11 $$Comm.num=\sum_{t=1}^{T}{cn}_{t}/NT$$

### Simulation parameters

To analyze the success of the different types of agents and how that depends on the different decision-making heuristics, we ran a series of independent and identical simulations of the model studying its evolutionary dynamics.

In the presented simulations, we have used the following parameters: $N=100$ (population size); $T=5\times$ ${10}^{6}$ (simulation length), $b=4$ (benefit), $c=1$ (cost); at the beginning of the simulations all the relationships are fixed with ${s}_{x,y}=0$, and ${s}_{x,x}=1$; $r=0.3$ (amount of relationship change); $\mu =0.01$ (mutation probability); $\beta =5$ (temperature parameter); $i=10$ (iteration frequency).

### Supplementary Information

Supplementary Information.

## Data Availability

The data and code supporting the findings of this study are openly accessible through the following repository: https://1drv.ms/u/c/f4e380fae79dcc18/EcFsrn-_7jFPuJq6R-pHZlkBxj8CbjAIQIv4GC3K7bXpwg or can be requested to the corresponding author.
