# Supplementary material for: Cooperation and social organization depend on weighing private and public reputations
Source: Sci Rep. 2024 Jul 16;14:16443. doi: 10.1038/s41598-024-67080-z (PMC11252375; doi:10.1038/s41598-024-67080-z)
Supplement: Supplementary file 1 — Supplementary Information. [file 41598_2024_67080_MOESM1_ESM.pdf]

Cooperation and Social Organization depend on Weighing Private and Public Reputations
SUPPLEMENTARY INFORMATION

Matteo Cavaliere, Guoli Yang, Carsten De Dreu, Jörg Gross

1 The Robustness of Evolutionary Dynamics - Different Reputation Changes

For a homogeneous population, all agents are of the same type. To check the robustness of the evolutionary dynamics in a homogeneous population composed by F and H agents, we tested two different values for the amount of reputation change ($r = 0.5$ and $r = 0.7$). Three combinations of private and public information are explored (as done in the main text), including prioritizing public information ($p = 0.2$ and $q = 0.8$), prioritizing private information ($p = 0.8$ and $q = 0.2$) and high trustiness ($p = 0.8$ and $q = 0.8$).

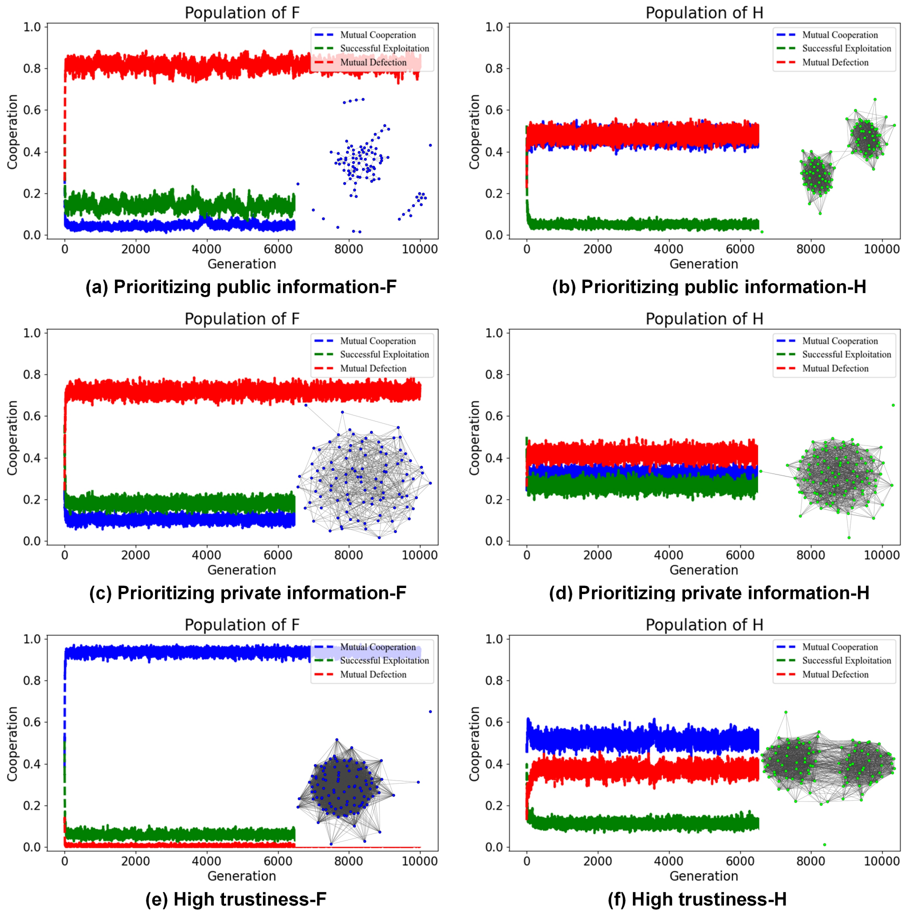

Figure S1: Trajectories and snapshots for the evolutionary dynamics with an intermediate value for the change of reputation $r = 0.5$. The size of the population is $N = 100$; iteration frequency is $i = 10$; mutation rate is $\mu = 0.01$. In each panel, we illustrate the proportion of actions of mutual cooperation, successful exploitation, and mutual defection.

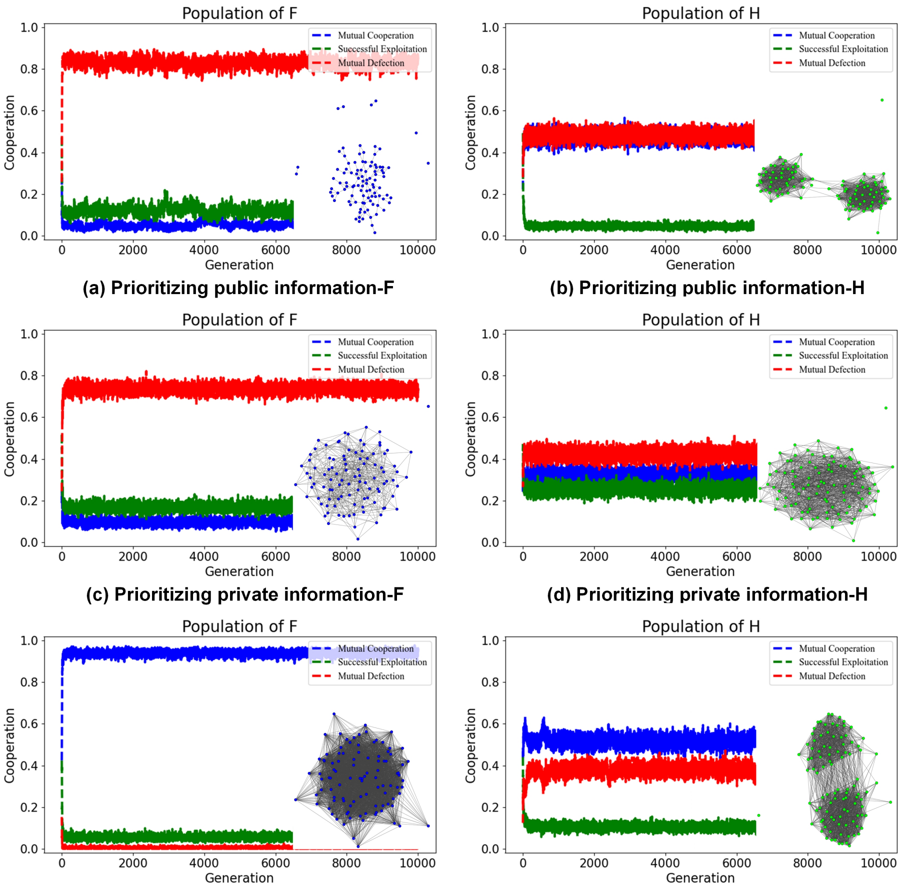

(a) Prioritizing public information-F (b) Prioritizing public information-H

(c) Prioritizing private information-F (d) Prioritizing private information-H

(e) High trustiness-F (f) High trustiness-H

Figure S2: Trajectories and snapshots for the evolutionary dynamics with a large value for the change of reputation $r = 0.7$. The size of the population is $N = 100$; iteration frequency is $i = 10$; mutation rate is $\mu = 0.01$. In each panel, we illustrate the proportion of actions of mutual cooperation, successful exploitation, and mutual defection.

As we can see from Figure S1 and S2, there is no obvious difference in cooperation and population structures by varying the value for the change of reputation r.

2 Pairwise Competition

2.1 Decision-making for Cooperation

We first consider the pairwise competitions of friend-focused agents against free-riders (namely FD competition), and those of Heider agents against free-riders (namely HD competition) to verify how successful friend-focused agents or Heider agents can cooperate with others of the same type and isolate the free-riders, under 6 different types of decisions (see the different cases presented in Figure 1 in the main text). Figure S3 presents the probabilities to cooperate or defect with same-type agents or free-riders.

For the FD competitions, friend-focused agents tend to cooperate more often with other friend-focused agents than with defectors, especially in the case of high trustiness (Figure S3(e)), where the proportion of case-e (both private and public information indicate to cooperate) is large. Furthermore, friend-focused agents frequently decide to (correctly) defect against defectors (based on decision represented by case-f).

For the HD competitions, Heider agents very often decide to cooperate with defectors (Figure S3(b) and (f)), mostly using case-c of the decision-making, where private information indicates to defect but public information indicates to cooperate, and the final decision is to cooperate. Heider agents can only successfully isolate defectors under prioritizing private information (Figure S3(d)), where Heider agents would more often choose to defect when public information indicates to cooperate and private information to defect (case-d).

We then consider pairwise competitions between friend-focused agents and Heider agents (namely FH competition) to verify how successful friend-focused agents or Heider agents can cooperate with those of same type and different type, under 6 different cases of decisions. Figure S4 presents the probabilities to cooperate or defect with each other.

In the case of prioritizing public information, friend-focused agents and Heider agents perform similarly (Figure S4(a) and (b)).

In the case of prioritizing private information, friend-focused agents are more likely to defect with others because of the decision associated to case-f (Figure S4(c)), but Heider agents prefer to defect under case-d. In addition, Heider agents have a relatively high probability to cooperate (Figure S4(d)), leading to the prevalence of Heider agents (Figure 6 in the main text).

In the case of high trustiness, cooperative friend-focused communities are formed with a high probability (Figure S4(e)). Heider agents, on the other hand, do not survive against friend-focused agents (Figure S4(f)), revealing that the enemy heuristics make the trust in the public reputation information exploitable.

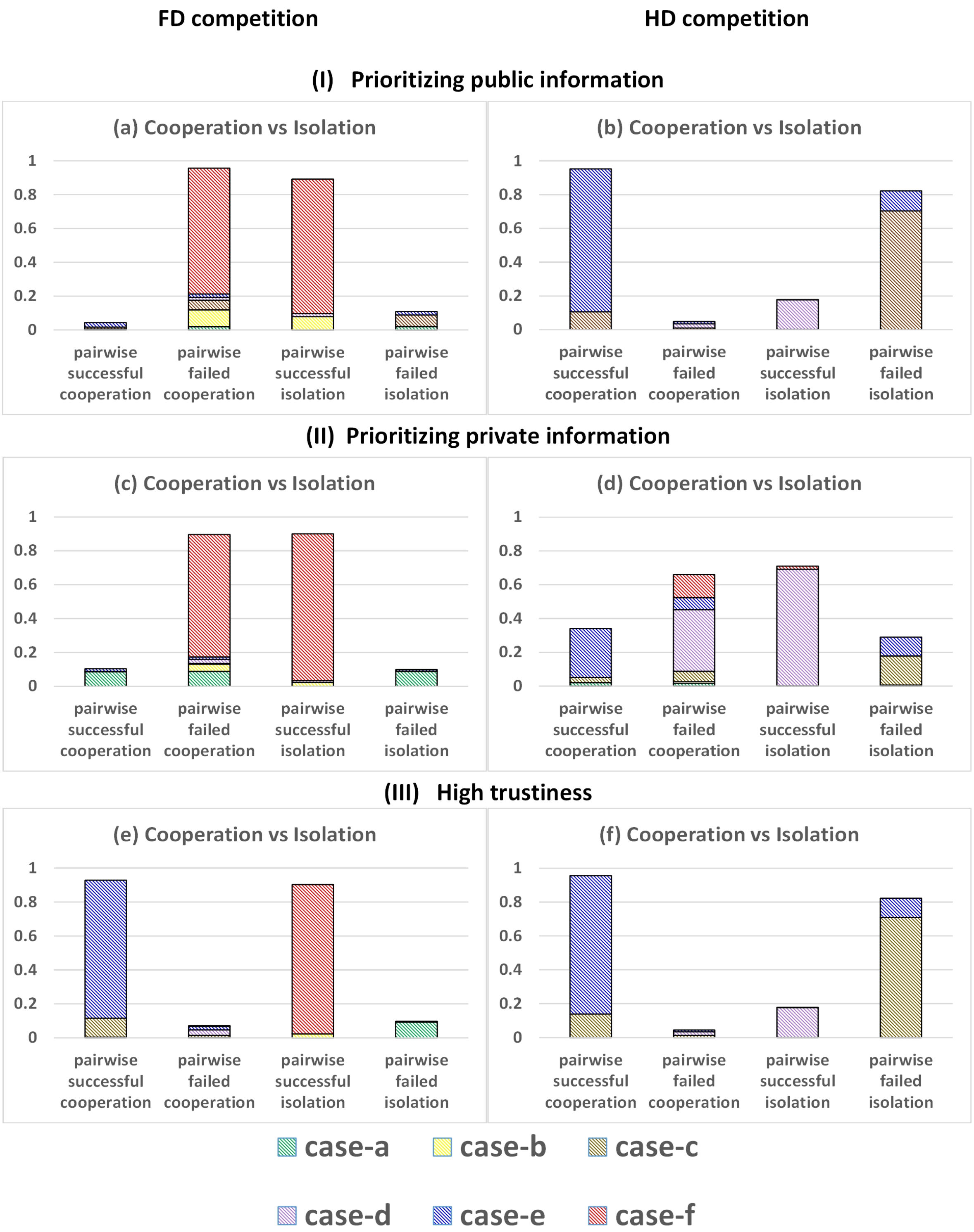

Figure S3: Probability of taking a certain decision by friend-focused agents (left panels) and Heider agents (right panels) in the pairwise competitions against free-riders. Three scenarios are considered, namely prioritizing public information (upper panels), prioritizing private information (middle panels) and high trustiness (lower panels), which are obtained by starting in a population with 50% of the two strategies considered. Each decision is sub-divided according to the proportion of the specific cases (see Fig 1 main text). Averages are obtained by running 10 independent simulations and each lasts 5×10^6 steps.

2.2 Population Metrics

To further illustrate the evolutionary dynamics by varying the strengths of public information and private information, we conduct some further experiments to present in more details some of the recorded statistical properties. Figure S5 presents the long-term averages of various population metrics recorded for different

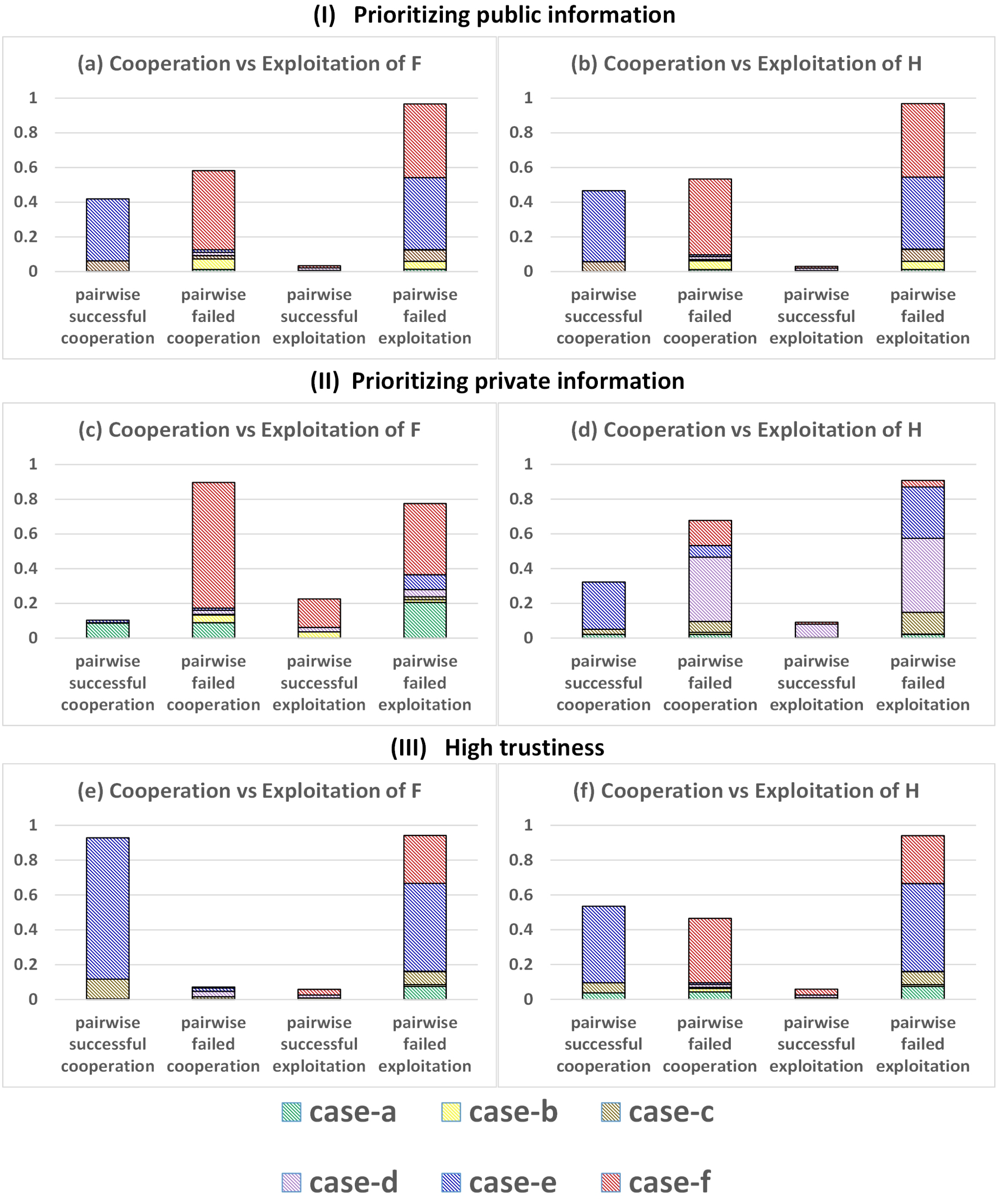

Figure S4: Probability of taking a certain decision by friend-focused agents (left panels) and Heider agents (right panels) in FH pairwise competitions. Three scenarios are considered, namely prioritizing public information (upper panels), prioritizing private information (middle panels) and high trustiness (lower panels), which are obtained by starting in a population with 50% of the two strategies considered. Each decision is sub-divided according to the proportion of the different cases (see Fig 1 main text). Averages are obtained by running 10 independent simulations and each lasts 5×10^6 steps.

public information strengths (q), for weak and strong private information (p).

We can observe that friend-focused agents outcompete Heider agents and free-riders for strong private and public information leading to a regime of highly cooperative, connected and prosperous population (Figure S5(b),(d)).

Free-riders can outcompete both Heider and friend-focused agents only when private information is weak and at an intermediate strength of public information (Figure S5 (c),(e)).

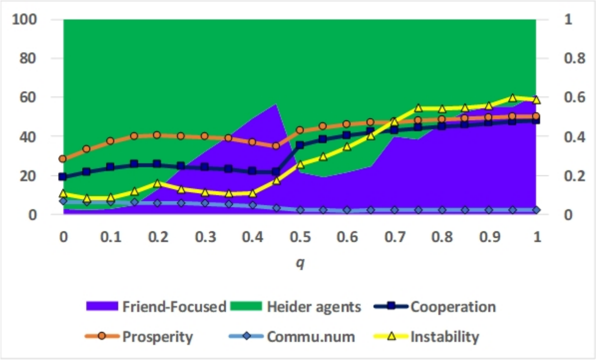

(a) weak private information for FH

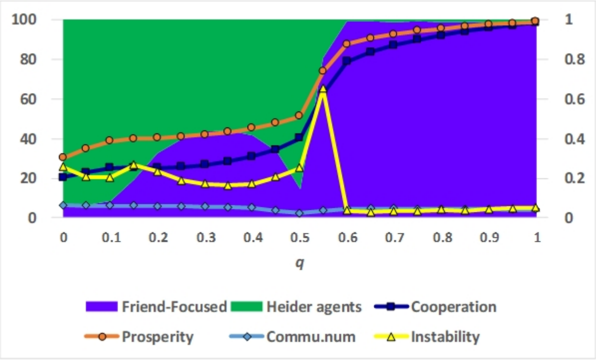

(b) strong private information for FH

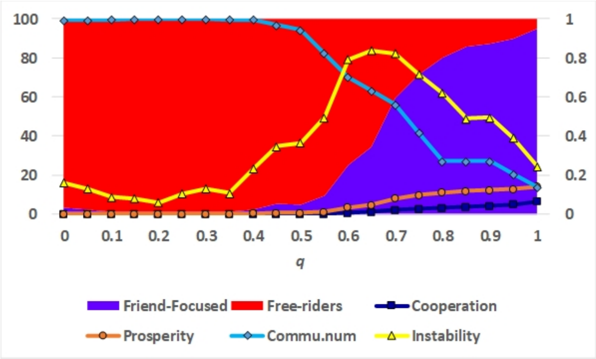

(c) weak private information for FD

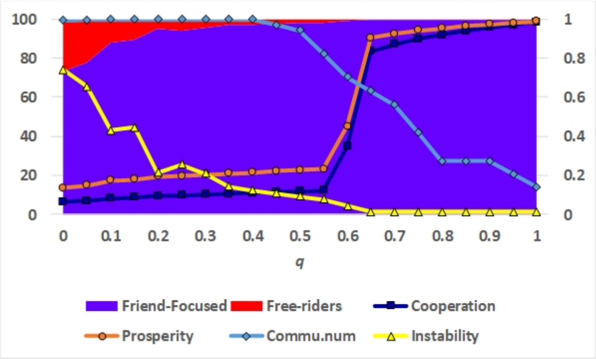

(d) strong private information for FD

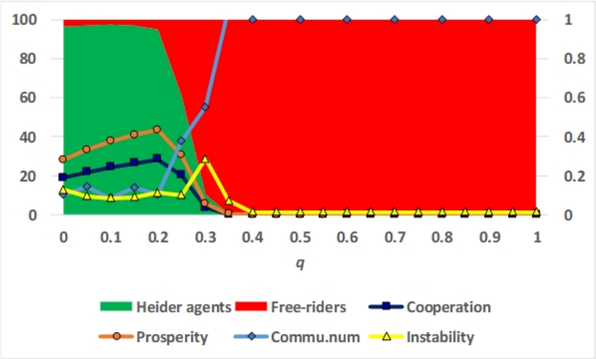

(e) weak private information for HD

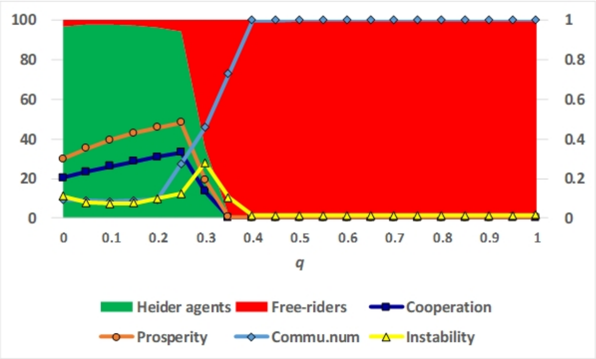

(f) strong private information for HD

Figure S5: Long-term averages of population composition, cooperation and prosperity for pairwise competitions (HD, FD, and FH) for weak ($p = 0.2$) and strong ($p = 0.8$) private information. Pairwise competitions (HD, FD, and FH) are obtained by starting with 50% of the two strategies considered. Averages are obtained by running 10 independent simulations and each lasts 5×10^6 steps.

Free-riders are outcompeted by Heider agents when public information is weak (Figure S5(e),(f)), and from friend-focused agents when public information is strong (Figure S5 (c),(d)).

Figure S6 presents the long-term average proportions of different agents, mutual cooperative actions, prosperity, instability and number of communities recorded for a fixed q and by varying qs, considering pairwise competitions.

In the case of competition FH, Heider agents are the majority of the population in the case of weak public information (Figure S6(a)), but they are outcompeted by the opponents for strong public information, especially when the strength of private information is large (i.e., large values of p and q), shown in Figure S6(b).

In the case of competition FD, free-riders form a stable population with low prosperity for weak public and private information (Figure S6(c)), but friend-focused agents become the dominant type for strong public information, especially when the strength of private information is large (Figure S6(d)).

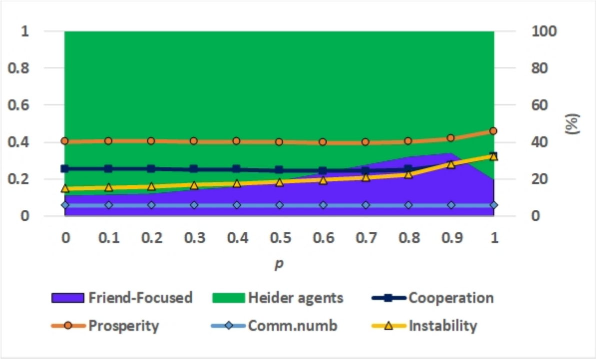

(a) weak public information for FH

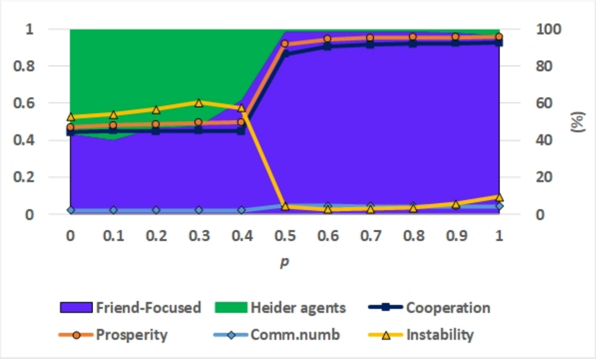

(b) strong public information for FH

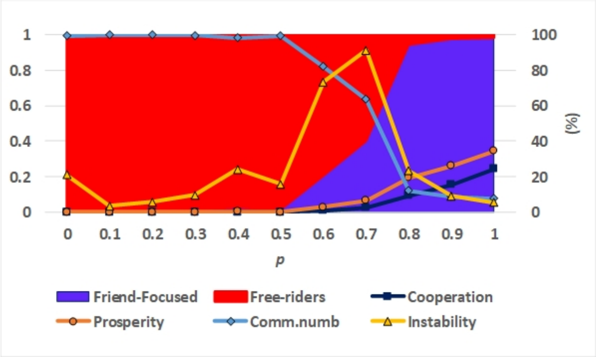

(c) weak public information for FD

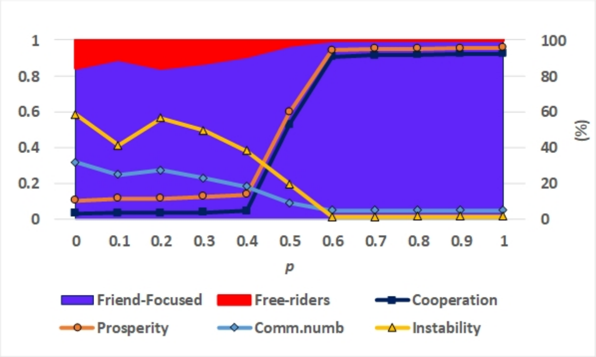

(d) strong public information for FD

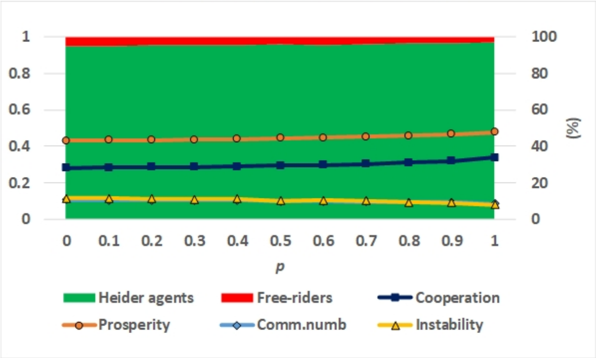

(e) weak public information for HD

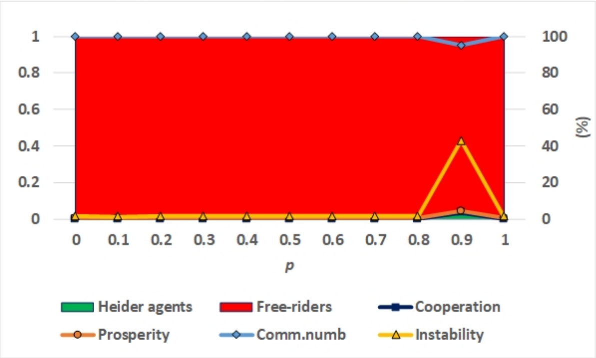

(f) strong public information for HD

Figure S6: Long-term averages of population composition, cooperation and prosperity for pairwise competitions (HD, FD, and FH) at weak ($q = 0.2$) and strong ($q = 0.8$) strength of public information. Pairwise competitions (HD, FD, and FH) are obtained by starting with 50% of the two strategies considered. Averages are obtained by running 10 independent simulations and each lasts 5×10^6 steps.

In the case of competition HD, the changes of the private information strength p only minimally affect the evolutionary dynamics – Heider agents dominate for weak public information (Figure S6(e)), and free-riders dominate for strong public information (Figure S6(f)).

3 Limit Scenarios

In this Section, we focus on four cases of decision-making which can be considered as limit scenarios of the scenarios considered in the main text. These scenarios generally reflect the same conclusions already discussed in the main text.

- Zero trustiness. This scenario corresponds to $p = 0$ and $q = 0$; if there is a conflict between the indication provided by public information and the one provided by private information, the agent will always choose to defect;
- P-precedence. This scenario corresponds to $p = 1$ and $q = 0$; if there is a conflict between the indication provided by public information and the one provided by private information, the agent will always choose to follow the decision provided by private information;
- Q-precedence. This scenario corresponds to $p = 0$ and $q = 1$; if there is a conflict between the indication provided by public information and the one provided by private information, the agent will always choose to follow the decision provided by public information;
- Fully Trusting. This scenario corresponds to $p = 1$ and $q = 1$; if there is a conflict between the indication provided by public and private information, the agent will always choose to cooperate.

We show in Figure S7 how the amount of cooperative actions, the stability and the organization of the population depends on the decision-making of the agents. We can observe that in the case of zero trustiness, the population is stable, with a dominance of Heider agents. Cooperation remains at low levels and the population is organized in a sparse network with a low number of positive links (Figure S7(a)).

In the P-precedence scenario ($q = 0, p = 1$), in case of conflict between the indication provided by public and private information, the agent follows the indication of the private information. In this case, there is no difference between friend-focused agents and Heider agents, and, as expected, they dominate the population alternately (Figure S7(b)).

In the case of Q-precedence ($p - 0$, $q = 1$), however, a mixed population with friend-focused agents (F) and Heider agents (H) will be invaded by free-riders (D), leading to the typical pattern of F-H-D cycles with high instability and intermediate levels of cooperation (Figure S7(c)).

In the case of the fully trusting scenario ($p = 1$ and $q = 1$), a densely connected population is formed with a stable dominance of friend-focused agents, where the free-riders never invade successfully (Figure S7(d)).

As we can see in Figure S7 both zero trustiness and fully trusting scenarios lead to a stable population with the dominance of Heiders and friend-focused agents, respectively. However, for P-precedence and Q-precedence scenarios, the population is unstable - this is caused by the growth of friend-focused agents coupled to the growth of a highly connected population (Figure S7(b),(c)).

Differently from the Q-precedence scenario, in the P-precedence scenario, there is no complete invasion of free-riders, friend-focused agents and Heiders dominate the population in alternative stages (Figure S7(b)).

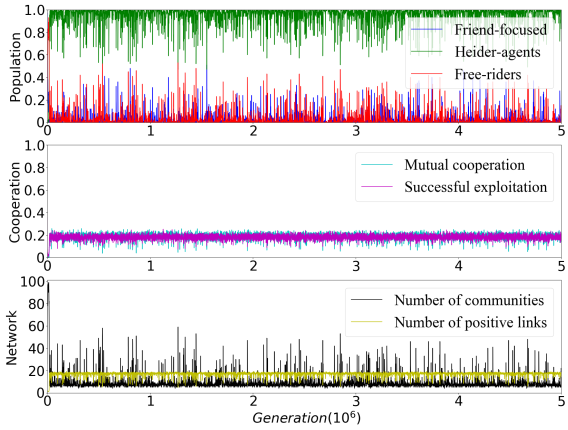

(a) Zero trustiness

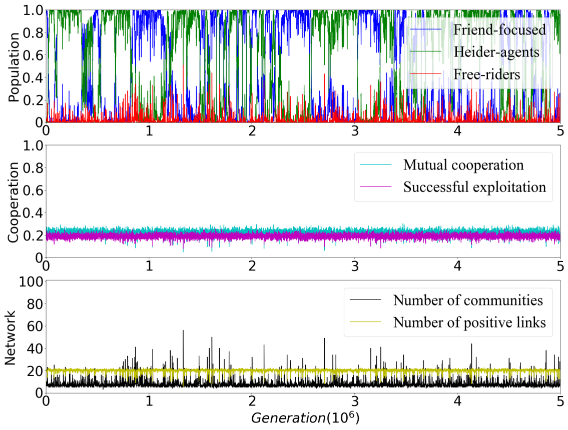

(b) P-precedence

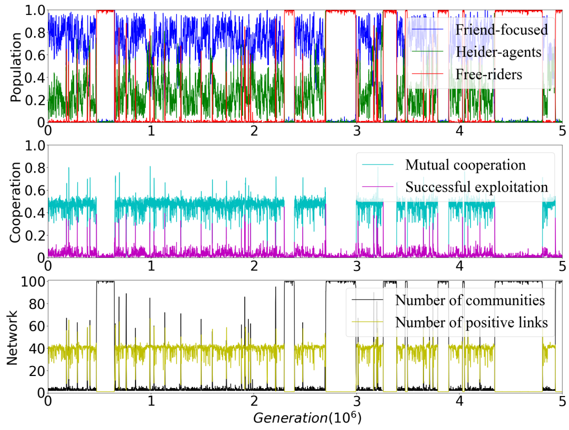

(c) Q-precedence

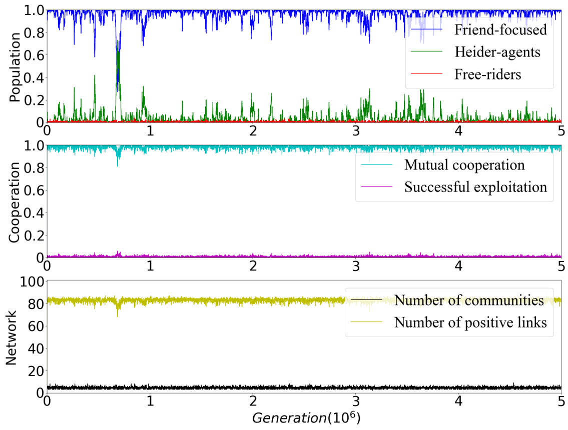

(d) Fully Trusting

Figure S7: Trajectories of the evolutionary dynamics in the case of zero trustiness, P-precedence, Q-precedence, and fully trusting. The size of the population is $N = 100$, iteration frequency is $i = 10$, mutation rate is $\mu = 0.01$, and the change of reputation $r = 0.3$. In each panel, we illustrate the proportion of friend-focused agents (blue curves), Heider agents (green curves), free-riders (red curves), fraction of cooperation actions (cyan curves), exploitative actions (magenta curves), number of communities (black curves) and positive links (yellow curves). In both zero trustiness and fully trusting scenarios we observe a stable population with the dominance of Heiders and friend-focused agents, respectively. Free-riders can more easily invade a mixed population of Heiders and friend-focused agents in the case of Q-precedence.

4 Steady State Distributions

For different values of p and q, the steady state distributions of the population are presented in Figure S8. Three cases are considered as follows: prioritizing public information ($p = 0.2$ and $q = 0.8$); prioritizing private information ($p = 0.8$ and $q = 0.2$); high trustiness ($p = 0.8$ and $q = 0.2$).

In the evolutionary dynamics, the population is dominated by different types of agents. When public information is prioritized, friend-focused agents perform better than Heider agents, and sometimes free-riders completely invade the population (Figure S8(a)). When private information is prioritized, the population is mostly composed by Heider agents and the free-riders cannot really invade (Figure S8(b)). For sufficiently large values of public and public information strengths, i.e., high trustiness, friend-focused agents dominate the population (Figure S8(c)).

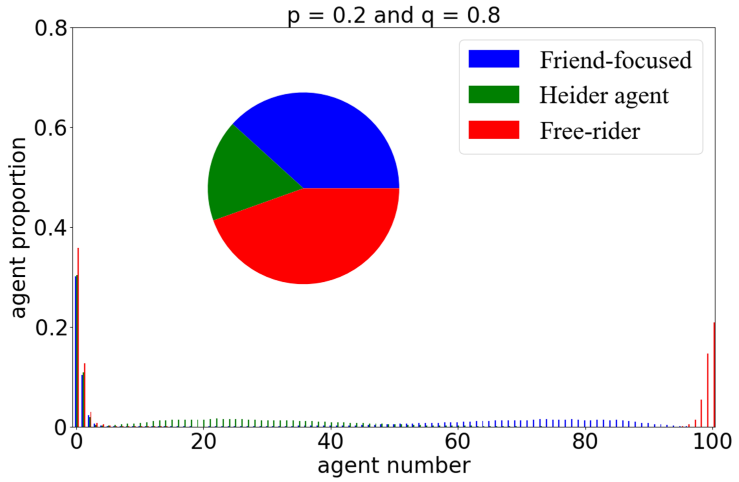

(a) Prioritizing public information

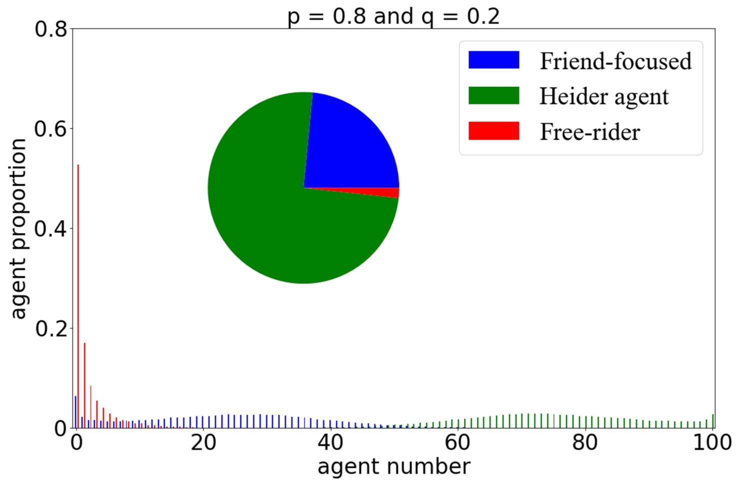

(b) Prioritizing private information

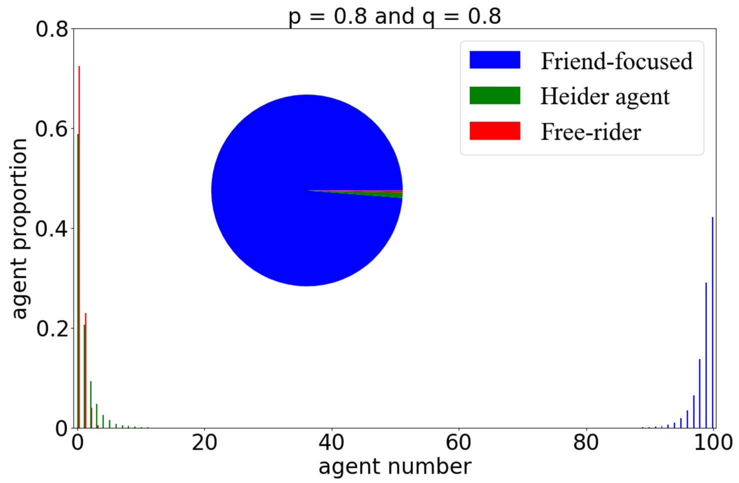

(c) High trustiness

Figure S8: Steady state distributions of the number of friend-focused agents, Heider agents and free-riders for different scenarios. The size of the population is $N = 100$; iteration frequency is $i = 10$; mutation rate is $\mu = 0.01$. The length of simulation is 5×10^6 steps.

5 Population Structure for Weak Private Information

We first analyze the scenario where the strength of the private information is small ($p = 0.2$). In this case, in Figure S9 we consider three values for the strength of the public information, $q = 0.2$ (left panels), $q = 0.5$ (middle panels) and $q = 0.8$ (right panels). Panels (a), (b) and (c) in Figure S9 illustrate the typical population structures obtained at different stages during the simulation, at $q = 0.2$, $q = 0.5$ or $q = 0.8$. In the presented snapshots, friend-focused agents are the blue nodes, Heider agents are the green nodes, and free-riders are the red nodes.

For a smaller value of q, the network is less connected and the population remains stable with a majority of Heider agents (Figure S9(a)). For larger values of q, the network is well connected and polarized with a mixed population of friend-focused agents and Heider agents (Figure S9(b),(c)).

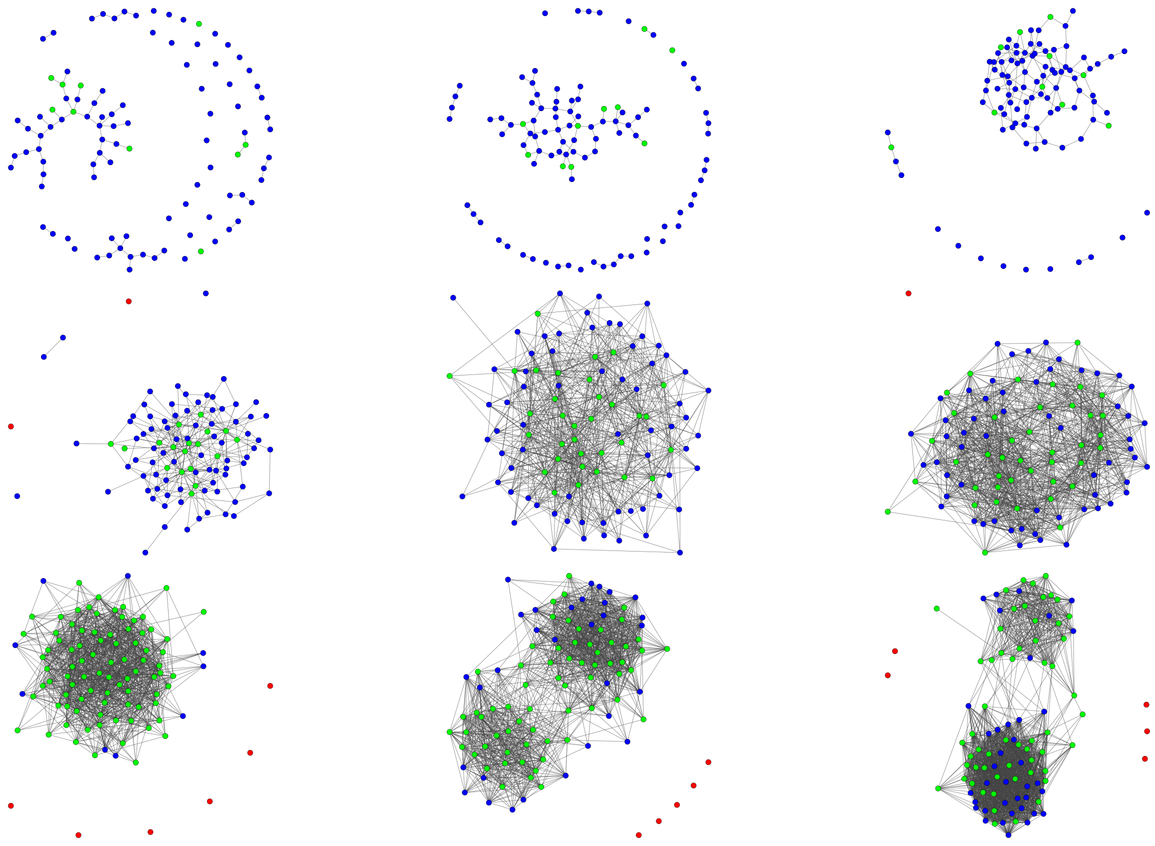

(a) q=0.2 (b) q=0.5 (c) q=0.8

Figure S9: Population structure for weak private information. Snapshots in the first row represent the typical networks observed at the beginning of the simulation where most of the population is composed by friend-focused agents; networks in the second row are composed by a similar amount of Heider and friend-focused agents; networks in the third row are composed by mostly Heider agents. We use weak private information ($p = 0.2$) and consider $q = 0.2$, $q = 0.5$ and $q = 0.8$ for the left, medium and right panels, respectively. The size of the population is $N = 100$; iteration frequency $i = 10$; mutation rate $\mu = 0.01$.

For intermediate values of the public information strength q, there are more severe invasions of free-riders that lead to the fall of cooperation (Figure S10). To reveal the underlying population structure associated with the invasion of free-riders and the following recovery of a mixed population composed by friend-focused agents and Heider agents, we illustrate in Figure S10 the snapshots of the typical networks observed at different stages of the evolutionary dynamics.

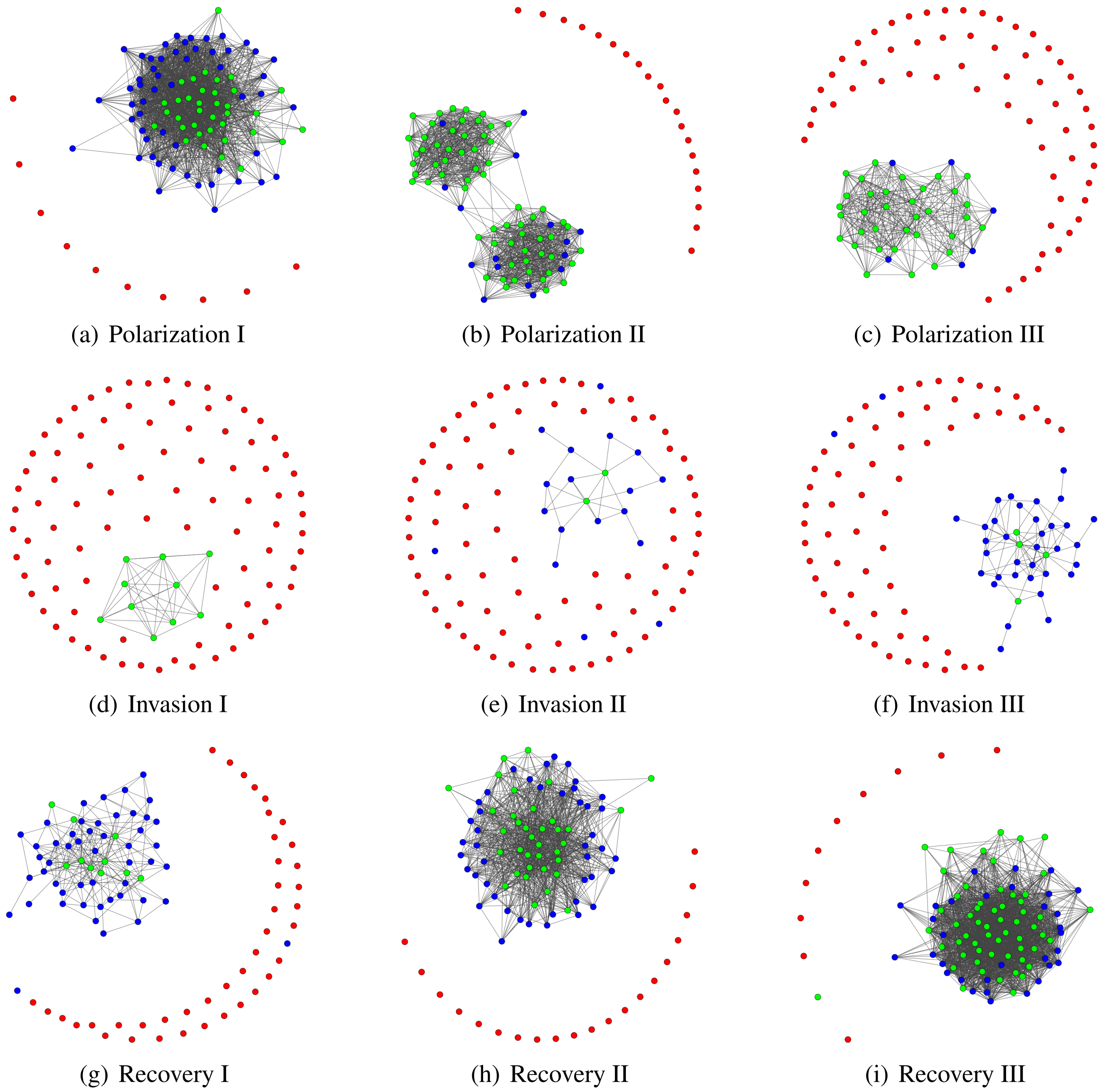

Figure S10: Temporary invasion of free-riders into a mixed population of friend-focused and Heiders agents for weak private information. The population becomes polarized (with mostly Heider agents - shown in green) just before the invasion of free-riders (red), where the population is fragmented into isolated nodes; after that phase, friend-focused agents (blue) start emerging and the population recovers to a mixed configuration. The snapshots are obtained for $p = 0.2$ and $q = 0.5$. The size of the population is $N = 100$; iteration frequency is $i = 10$; mutation rate is $\mu = 0.01$.

6 Population Structure for Strong Private Information

In this Section, we analyze the case in which the strength of the private information q is large ($p = 0.8$). When the strength of private information is large ($p = 0.8$), the evolution of the population is shown in Figure S11. In this case, we consider three values of the public information strength q: $q = 0.2$ (left panels), $q = 0.5$ (middle panels) and $q = 0.8$ (right panels) and show the consequences on the population structure. For a small value of q, the network is less connected and the population remains stable with a majority of Heider agents (Figure S11(a)). For an intermediate value of q, the network is well connected, and polarization is formed with a mixed population of friend-focused agents and Heider agents (Figure S11(b)). For large values of q (e.g., $q = 0.8$) the network is highly connected, and the population is relatively stable with a majority of friend-focused agents - invasions of free-riders are rarely successful (Figure S11(c)).

For intermediate values of the public information strength q, there are more frequent invasions of free-riders that lead to the fall of cooperation (Figure S12). The underlying population structure associated with

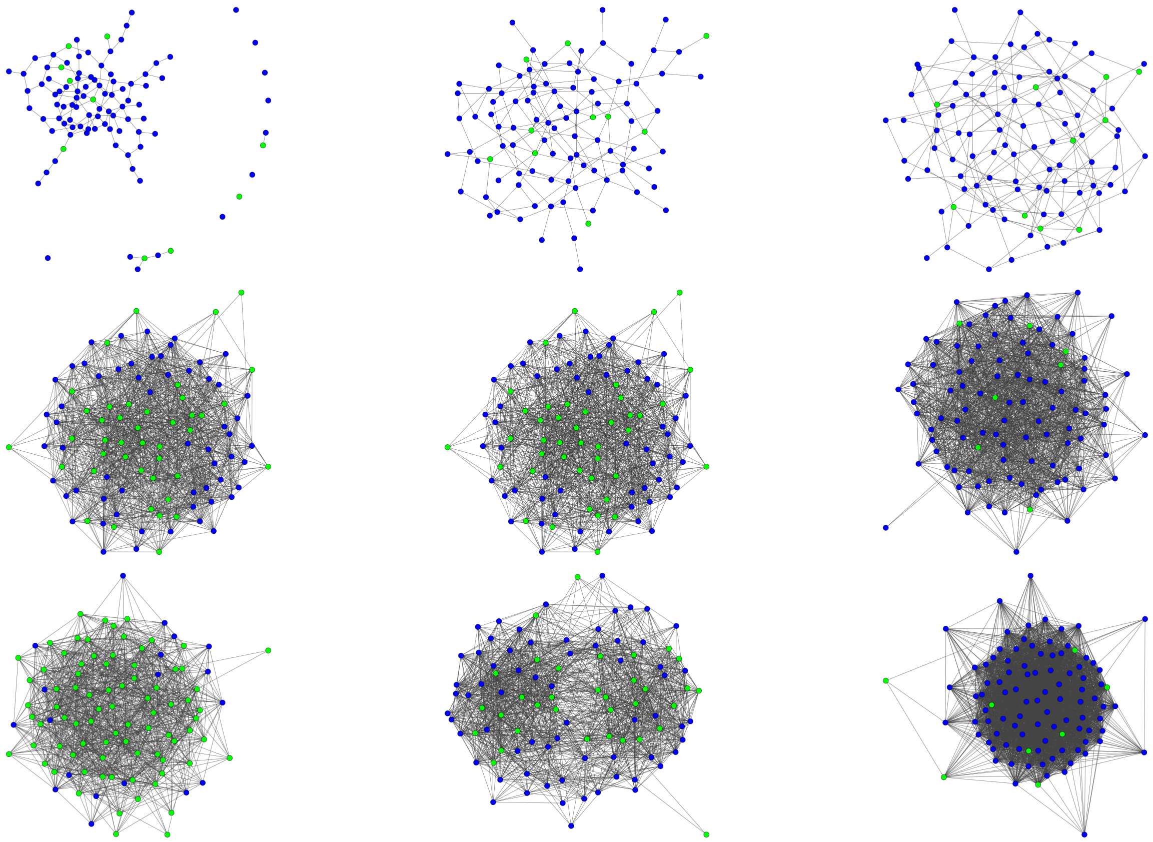

(a) q=0.2 (b) q=0.5 (c) q=0.8

Figure S11: Population structure for strong private information. The snapshots show the typical observed networks. We use a large strength for private information ($p = 0.8$) and consider $q = 0.2$, $q = 0.5$ and $q = 0.8$ for the left, middle and right panels, respectively. The snapshots present the population evolving from an initial sparse graph to a stable population of Heider agents (at $q = 0.2$), to a polarized population (at $q = 0.5$), and to a stable population of friend-focused agents (at $q = 0.8$). The size of the population is $N = 100$; iteration frequency $i = 10$; mutation rate $\mu = 0.01$.

the invasion of free-riders and the following recovery of a mixed population composed by friend-focused agents and Heider agents, is presented in Figure S12 which shows the snapshots of the typical networks observed at different stages of the evolutionary dynamics.

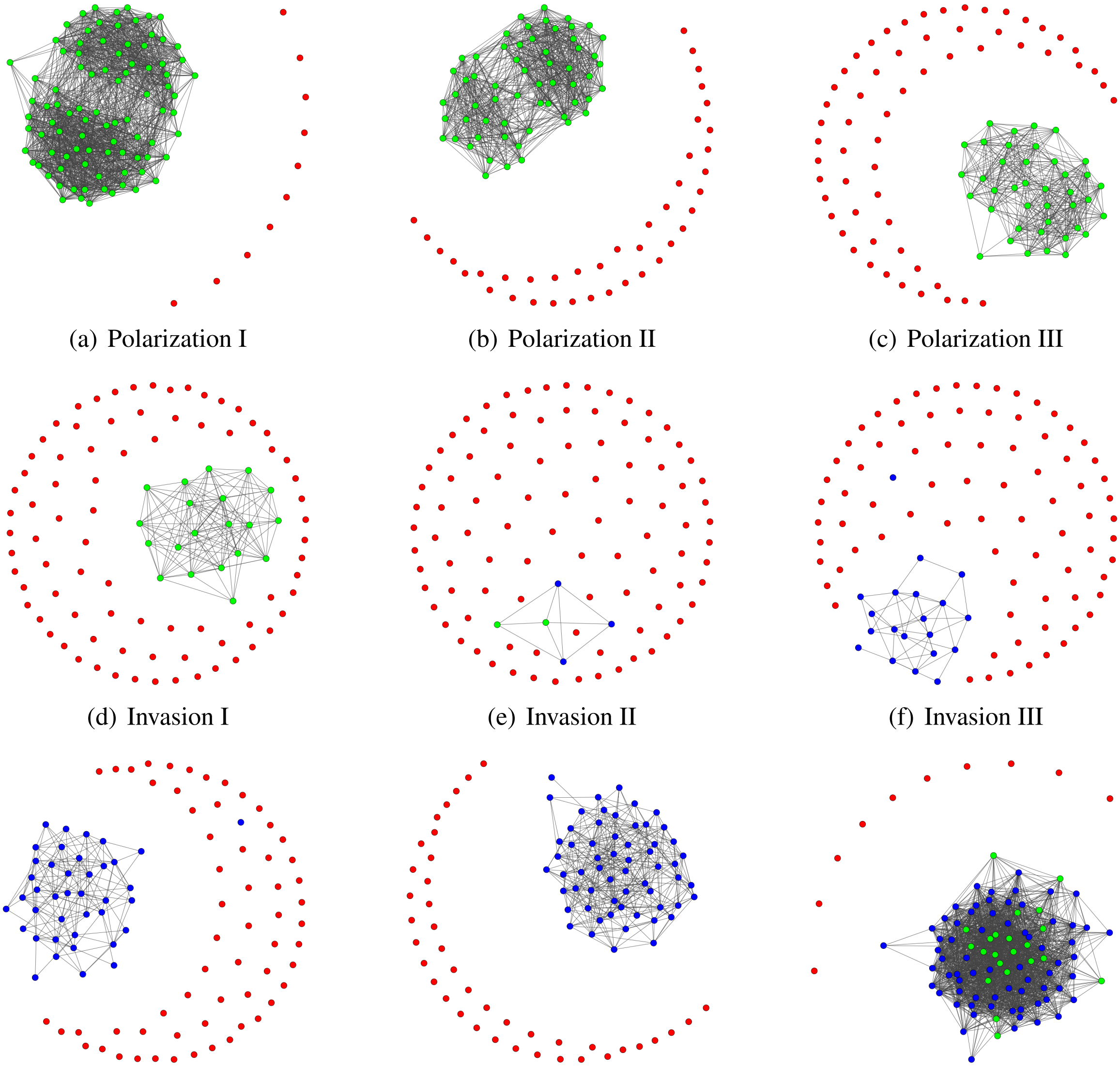

(a) Polarization I (b) Polarization II (c) Polarization III

(d) Invasion I (e) Invasion II (f) Invasion III

(g) Recovery I (h) Recovery II (i) Recovery III

Figure S12: Temporary invasion of free-riders into a mixed population of friend-focused and Heiders agents for strong private information. We show the population in the stage of polarization, invasion and recovery. The polarization of the population (mainly consisting of Heider agents, shown in green) is formed just before the invasion of free-riders (red), with the population fragmented into isolated nodes; after that phase, friend-focused agents (blue) start emerging and the population recovers to a mixed composition. The snapshots are obtained for $p = 0.8$ and $q = 0.5$. The size of the population is $N = 100$; iteration frequency is $i = 10$; mutation rate is $\mu = 0.01$.

7 Decision-Making, Transitions and Evolutionary Dynamics For Weak and Strong Private Information

To understand the long-term dynamics of the population for weak and strong private information, we conduct a series of simulations to obtain the average values of the population composition, the network structure and agents'decisions; we consider a variety of decision-making by fixing the strength of public information q, and varying the strength of private information p.

In Figure S13 we plot the long-term average values of a variety of statistical properties, including the proportion of different types of agents in the population, cooperation level, number of communities, average population payoff (indicating the prosperity of the population), and population instability calculated using the standard deviation of agents' population size (as defined in the main text).

By fixing the strength of private information and varying the strength of public information, we can

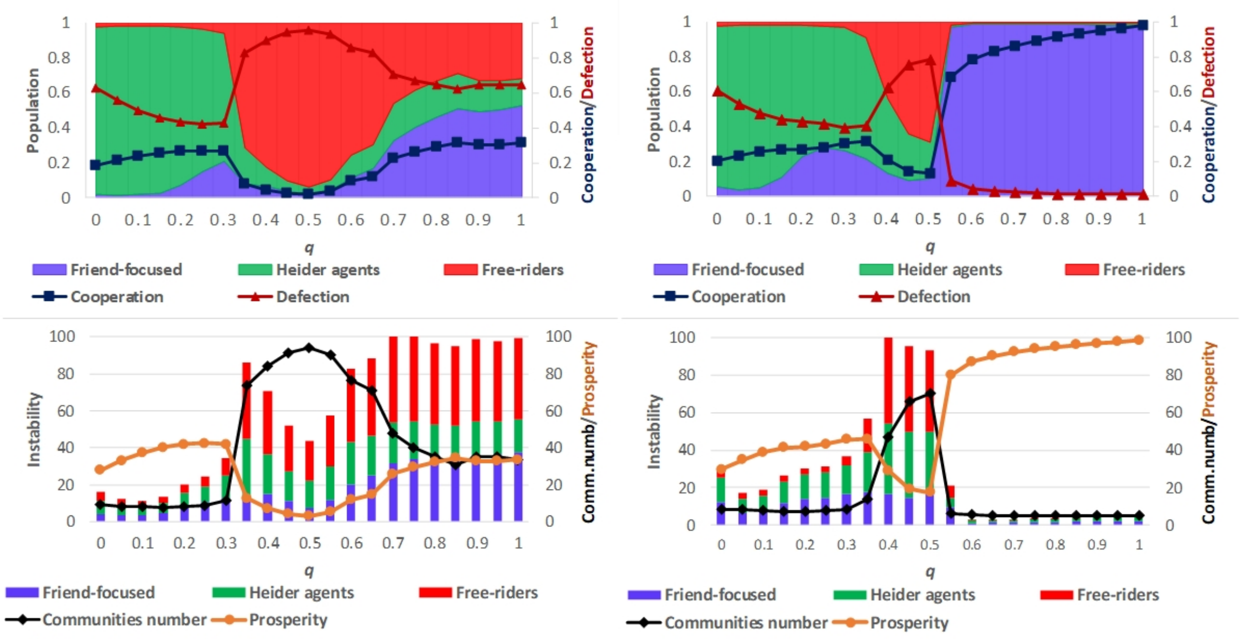

(a) weak private information **(b) strong private information**

Figure S13: Long-term average values of population composition, cooperation, instability and prosperity for (a) weak private information ($p = 0.2$) and (b) strong private information ($p = 0.8$). For each panel, parameter q is varied between 0 and 1, and we compute various statistical properties. The proportion of the different types of agents are illustrated in the first row by area of blue, green and red; the two curves overlapped show the proportions of cooperation and defection. For the second row we use color bars to show the instability; the two curves overlapped show the number of communities and prosperity. Averages are obtained by considering 10 independent simulations, each one lasting 5×10^6 steps.

observe a peak of instability and defection for an intermediate value of public information strength. In the case of weak private information (Figure S13(a)), we can also observe that before the increase of defection, the population instability increases (around $q = 0.35$), and decreases again when the population is composed by mostly cheaters (around $q = 0, 5$); as the strength of public information q is further increased the population instability increases again, with more frequent (temporary) invasions of free-riders, leading to the observed cycles of F-H-D. When the strength of private information is large (Figure S13(b)), a low strength for public information q facilitates the dominance of Heider agents. Similarly, for intermediate values of qs, we can observe an increase in the amount of free-riders, associated with high population instability. However, a further increase of q, leads to the dominance of friend-focused agents associated with a stable and prosperous population.

8 Decision-Making, Transitions and Evolutionary Dynamics for Weak and Strong Public Information

To better understand the long-term dynamics of the population for weak and strong public information, we conduct a series of simulations to compute the average values of the population composition, the network structure and agents' decisions; we consider a variety of decision-making by fixing the strength of public information q, and varying the strength of private information p.

In Figure S14 we plot the long-term average values of a variety of statistical properties, including the proportion of different types of agents in the population, cooperation level, number of communities, average population payoff (indicating the prosperity of the population), and population instability calculated using the standard deviation of agents' population size (defined in the main text).

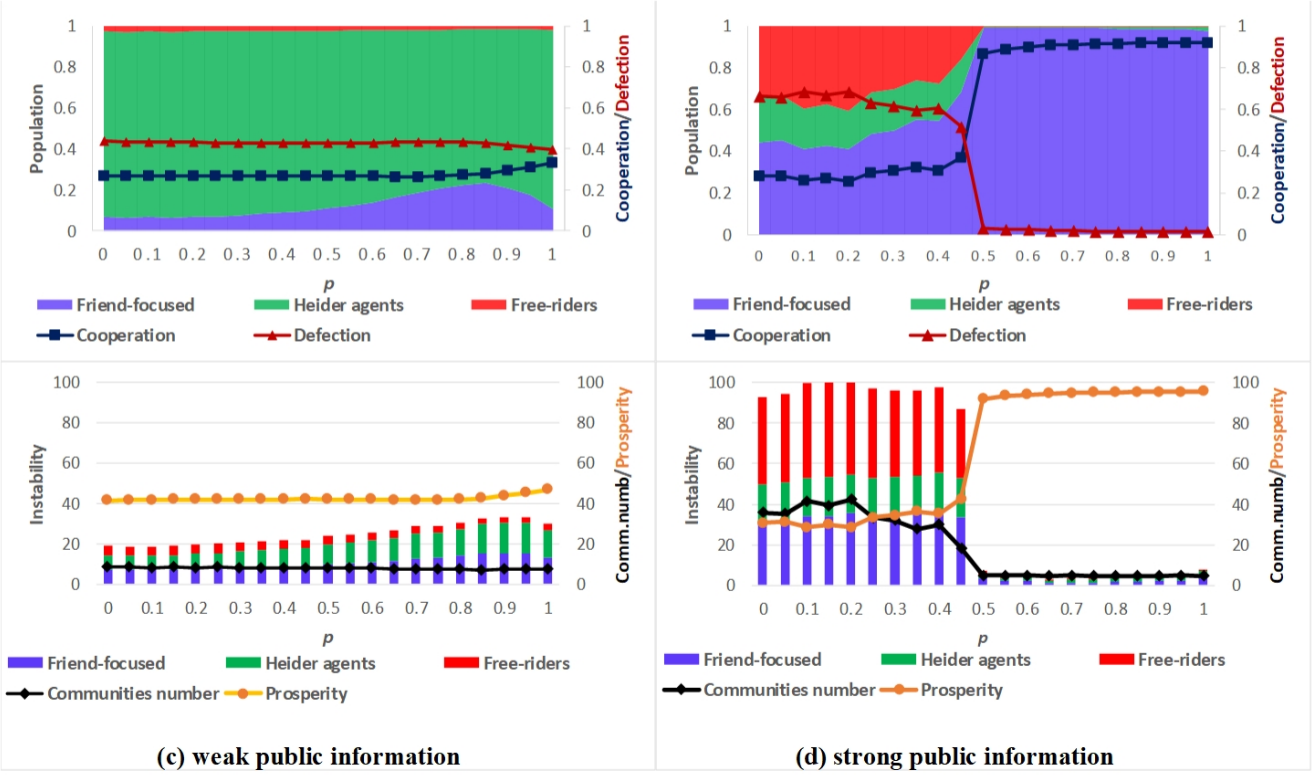

Figure S14: Long-term average values of population composition, cooperation, population instability and prosperity for (a) weak public information ($q = 0.2$), (b) strong public information ($q = 0.8$). For each panel, p is varied between 0 and 1, and we compute various statistical properties. The proportions of different types of agents are illustrated in the first and third row (blue, green and red areas); the two overlapped curves show the proportions of cooperation and defection. For the second and fourth rows we use color bars to show the standard deviations (i.e., instability) of the population; the two overlapped curves show the number of communities and the average population payoff (i.e., prosperity). Averages are obtained by considering 10 independent simulations, each one lasting 5×10^6 steps.

When the strength of public information is small, invasions of free-riders are rare - population is generally dominated by Heider agents organized in a rather stable population with few communities (Figure S14(a)) for varying strengths of private information. For a large strength of public information, a sharp transition from an unstable population composed by a mix of free-riders, Heider and friend-focused agents, to a stable, well-connected and prosperous friend-focused component, can be observed at an intermediate private information strength (Figure S14(b)). After such point, mutual cooperation and population prosperity rise, and once a well connected population of friend-focused agents is formed, the population will remain stable by getting rid of free-riders and Heider agents.

9 Exploring Public and Private Information Strengths

As we have shown, the dominant strategy in the population is a consequence of the strengths of public and private information and it has effects on cooperation, population organization and overall prosperity.

To shed more light on the fine balance between public and private information, we have systematically varied the parameters p (representing the private information strength) and q (representing the public information strength) in Figure S15 and analyzed the long-term average of (a) the proportion of friend-focused agents; (b) proportion of Heider agents; (c) proportion of free-riders; (d) number of mutual cooperative actions; (e) number of exploitative actions; (f) number of mutual defective actions; (g) prosperity of the population (average payoff); (h) number of complete invasions of free-riders; (i) number of communities in the population. The number of complete invasions is computed by considering the number of transitions which start from a population without any free-rider and lead to a population composed by only free-riders. Intuitively, this measure describes the vulnerability of the population to free-riders.

Figure S15 has been obtained by starting with a population without free-riders, and using 10 independent simulations, each one lasting 5×10^6 iterations. In a single simulation, each $i = 10$ iterations, the population evolves according, as described in the main text, to the Moran process with a mutation rate $\mu = 0.01$.

As we can observe in Figure S15(a), (b) and (c), the dominant type of agents depend on the strengths of private information (p) and public information (q). The amount of friend-focused agents increases for stronger private and public information (large values of p and q), while Heider agents are dominant for weak public information ($q < 0.4$). We can also see that friend-focused agents dominate by choosing cooperation: in fact the regime of p and q where they dominate correlates with the one where cooperation and prosperity are maximal (Figure S15(a),(d) and (g)). On the other hand, we can say that Heider agents win by choosing exploitation: the regime of p and q where Heider agents dominate correlates with the area with highest exploitation, low cooperation and low prosperity (Figure S15(b), (d), (e) and (g)). Free-riders invade more frequently the population at intermediate values of public information (around $q = 0.5$) and weak private information (Figure S15(h)). This area is associated with a large number of mutually defective actions and communities numbers (Figure S15(f) and (i)), which indicate that the invasions of free-riders lead to fragmented populations. Isolated free-riders keep their dominance over the other types of agents, and this is associated to the fall of prosperity (Figure S15(g)). The strengths of public and private information have also an effect on the organization of the population. As we can see in Figure S15, the dominance of Heider agents is generally associated to more compact communities, with high levels of exploitation and defection leading to low prosperity. On the other hand, the dominance of friend-focused agents is generally associated to a more connected community correlated to high cooperation, minimal exploitation and high prosperity. The dominance of free-riders is associated to a more fragmented population structure (higher communities number), with frequent collapse and revival of free-riders invasions, and, as expected, high levels of defection and very low prosperity.

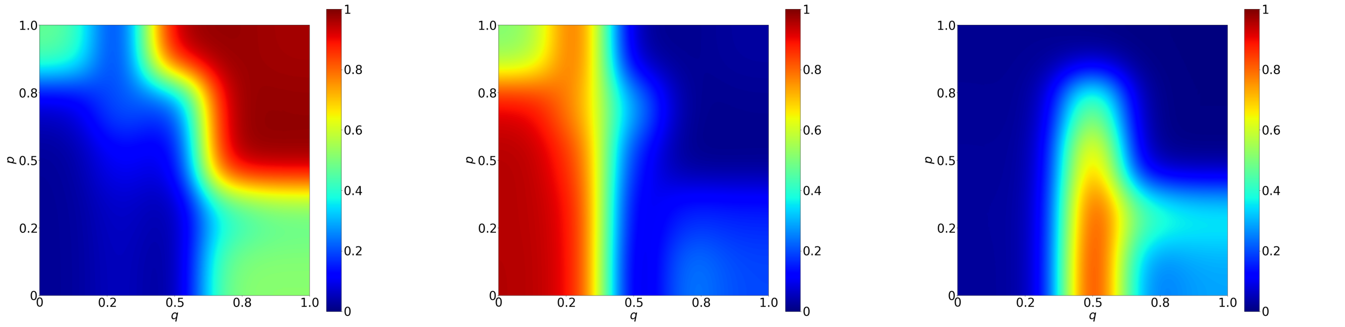

(a) Friend-focused (b) Heider agents (c) Free-riders

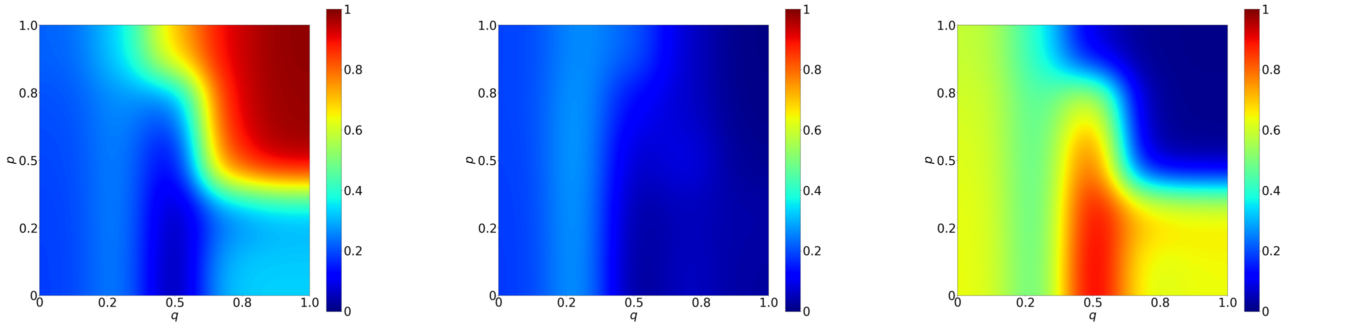

(d) Cooperation (e) Exploitation (f) Defection

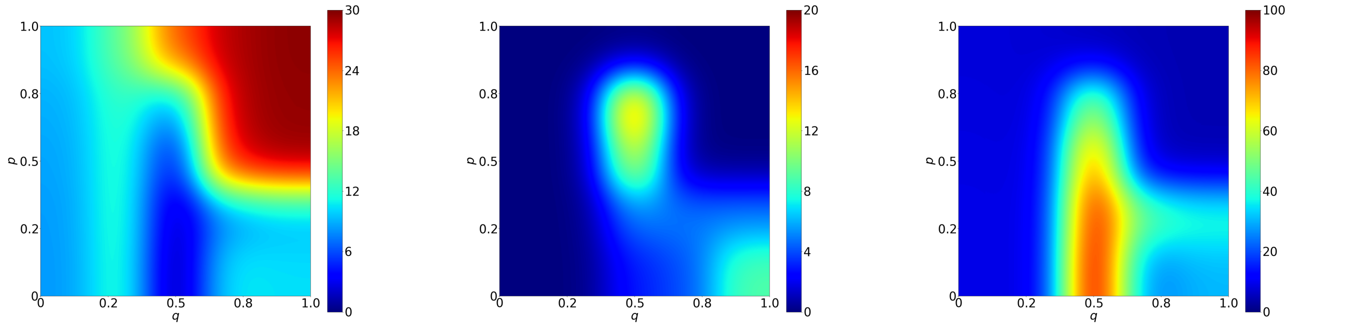

(g) Prosperity (h) Invasions of free-riders (i) Number of communities

Figure S15: Long-term averages of the fractions of different types of agents, prosperity, cooperation, number of communities and invasions for varying ps and qs. Friend-focused agents dominate the population for large values of p and q, along with the rise of cooperation and prosperity. Heider agents dominate the population for small values of q; their rise correlates with an increase in exploitation; free-riders invade more frequently at intermediate values of qs along with the rise of defection and communities number. Averages in the heatmap are obtained by running 10 independent simulations, each one lasting 5×10^6 steps.

10 Adding Enemy-Focused agents and Incomplete Heider agents

To further understand the ability of Heider rules to evolve and be resilient against free-riders (defectors), we consider two additional types of agents: enemy-focused agents and incomplete Heider agents, introduced in the SI of [1].

(1) Enemy-focused agents (denoted by E), only take weighted opinion of enemies into account, namely those with $s_{ij} < 0$, when they are computing the probability to cooperate given the available public information. In this case, an enemy-focused agent will implement the heuristic of 'a friend of an enemy is an enemy'

and ’an enemy of an enemy is a friend’.

In the pairwise competition between enemy-focused agents and free-riders, prioritizing private information leads to the success of enemy-focused agents (Figure S16). However, free-riders will dominate the population in the other scenarios.

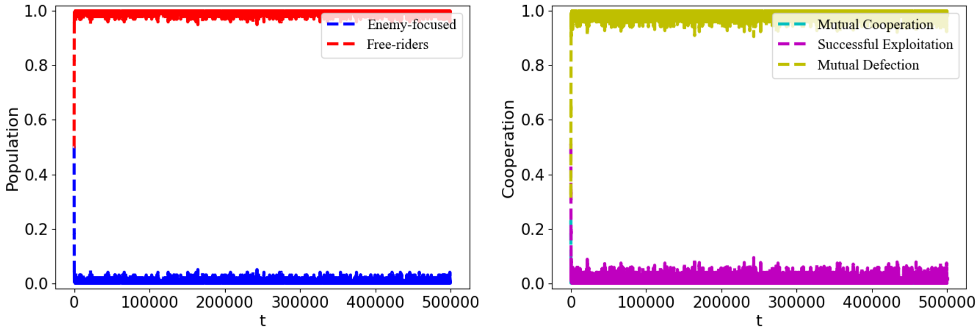

(a) Prioritizing public information

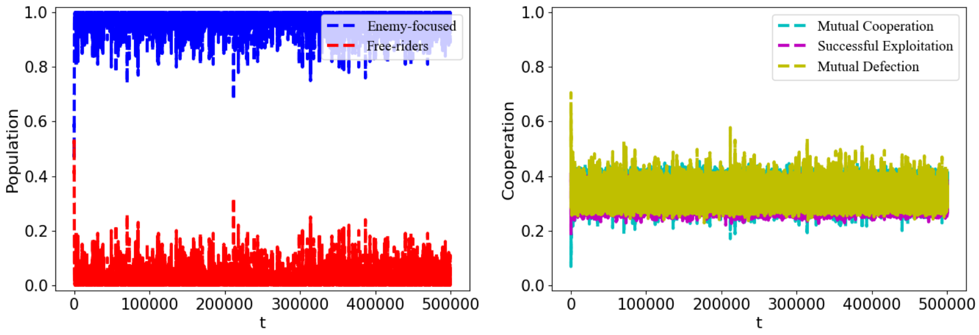

(b) Prioritizing private information

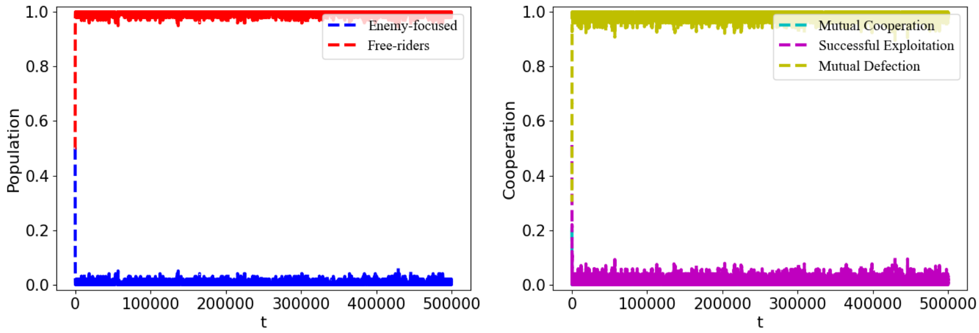

(c) High trustiness

Figure S16: Pairwise competition between enemy-focused agents and free-riders. The size of the population is $N = 100$, iteration frequency is $i = 10$, mutation rate is $\mu = 0.01$, and the change of reputation $r = 0.3$. In each left panel, we illustrate the proportion of enemy-focused agents (blue curves), free-riders (red curves). In each right panel, we illustrate the fraction of cooperation actions (cyan curves), exploitative actions (magenta curves), and defection actions (yellow curves).

(2) Incomplete Heider agents (denoted by I), implement the heuristic of ’a friend of a friend is a friend’, ’an enemy of a friend is an enemy’, and ’a friend of an enemy is an enemy’, but not the last heuristic ’an enemy of an enemy is a friend’.

In the pairwise competition between incomplete Heider agents and free-riders, we observe the dominance of free-riders in the three scenarios of prioritizing public information, prioritizing public information and high trustiness (Figure S17). However, the population is not stable when the strength of public information is large (Figure S17(a)(c)).

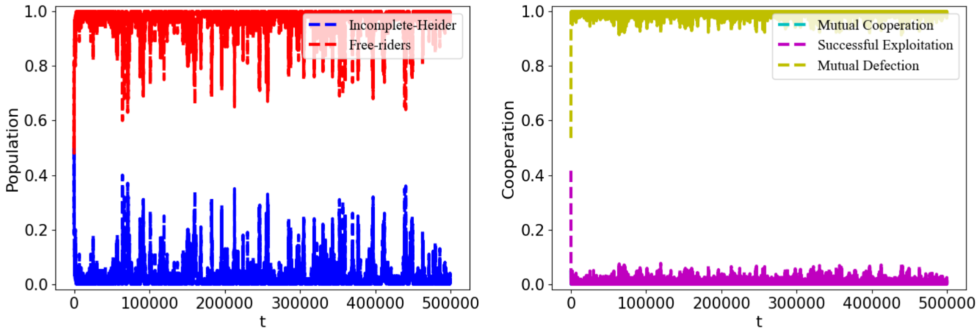

(a) Prioritizing public information

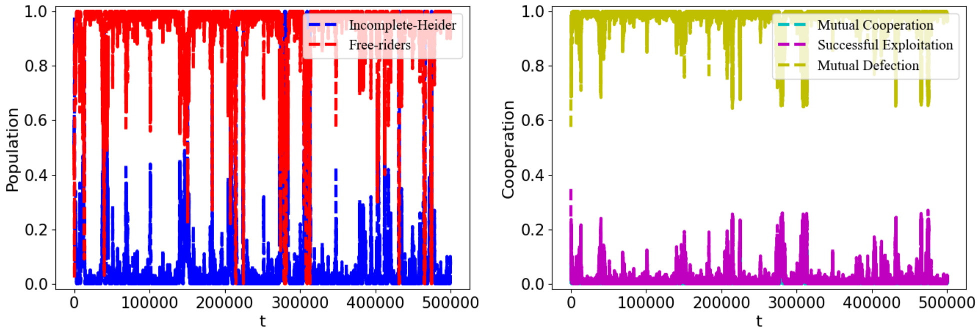

(b) Prioritizing private information

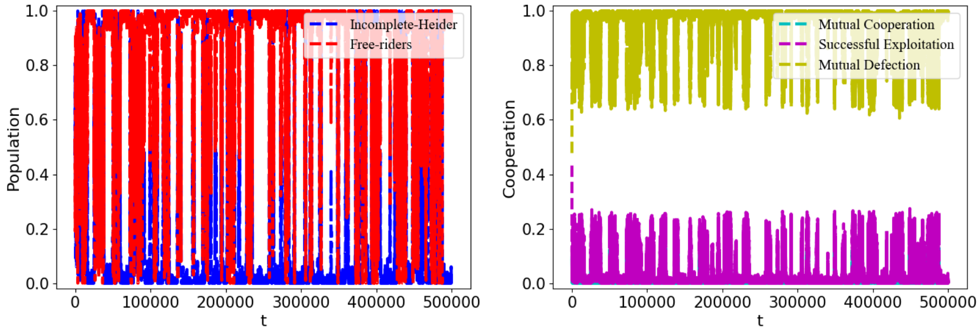

(c) High trustiness

Figure S17: Pairwise competition between incomplete Heider agents and free-riders. The size of the population is $N = 100$, iteration frequency is $i = 10$, mutation rate is $\mu = 0.01$, and the change of reputation $r = 0.3$. In every left panel, we illustrate the proportion of incomplete Heider agents (blue curves), free-riders (red curves). In every right panel, we illustrate the fraction of cooperation actions (cyan curves), exploitative actions (magenta curves), and defection actions (yellow curves).

We then consider all the remaining possibilities of pairwise competition and measure the success (win) or failure (lose) of a certain type of strategy by calculating the average number of agents with that type of strategy in the population. The type of strategy which is employed by the majority of the agents in the population is defined to be the winning type. Results are then summarized in Table 1. In the table, F denotes friend-focused agents, H denotes Heider agents, E denotes enemy-focused agents, I denotes incomplete Heider agents, and D denotes defectors (i.e., free-riders). Adding these new strategies does not change, however, the main results seen in the main text. In fact, we can see that in pairwise competitions, Heider agents win when private information is prioritized, friend-focused agents win the case of high trustiness. However, when public information is prioritized, there is no always-winning strategy.

(I) Prioritizing public information
($p = 0.2$, $q = 0.8$)

	F	H	E	I	D
F	\	lose	win	lose	win
H	win	\	lose	lose	lose
E	lose	win	\	lose	lose
I	win	win	win	\	lose
D	lose	win	win	win	\

(II) Prioritizing private information
($p = 0.8$, $q = 0.2$)

	F	H	E	I	D
F	\	lose	lose	win	win
H	win	\	win	win	win
E	win	lose	\	win	win
I	lose	lose	lose	\	lose
D	lose	lose	lose	win	\

(III) High trustiness
($p = 0.8$, $q = 0.8$)

	F	H	E	I	D
F	\	win	win	win	win
H	lose	\	lose	win	lose
E	lose	win	\	lose	lose
I	lose	lose	win	\	lose
D	lose	win	win	win	\

Table 1: Table representing the success or failure in pairwise competition of FH (friend-focused agents vs Heider agents), FE (friend-focused agents vs enemy-focused agents), FI (friend-focused agents vs incomplete Heider agents), FD (friend-focused agents vs free-riders), HE (Heider agents vs enemy-focused agents), HI (Heider agents vs incomplete Heider agents), HD (Heider agents vs free-riders), EI (enemy-focused agents vs incomplete Heider agents), ED (enemy-focused agents vs free-riders), ID (incomplete Heider agents vs free-riders). The size of the population is $N = 100$, iteration frequency is $i = 10$, mutation rate is $\mu = 0.01$, and the change of reputation $r = 0.3$.

References

1. Jörg Gross and Carsten KW De Dreu. The rise and fall of cooperation through reputation and group polarization. *Nature communications*, 10(1):1–10, 2019.
